# Reproducibility, Specificity and Accuracy of Relative Quantification Using Spectral Library-based Data-independent Acquisition*[Fn FN2]

**DOI:** 10.1074/mcp.RA119.001714

**Published:** 2019-11-07

**Authors:** Katalin Barkovits, Sandra Pacharra, Kathy Pfeiffer, Simone Steinbach, Martin Eisenacher, Katrin Marcus, Julian Uszkoreit

**Affiliations:** Ruhr University Bochum, Faculty of Medicine, Medizinisches Proteom-Center, Bochum, Germany

**Keywords:** Mass Spectrometry, Quantification, Label-free quantification, Bioinformatics software, Target identification, data-independent acquisition (DIA), peptide identification, proteomics, spectral library

## Abstract

Currently data-dependent acquisition (DDA) is the method of choice for mass spectrometry-based proteomics discovery experiments, but data-independent acquisition (DIA) is steadily becoming more important. One of the most important requirements to perform a DIA analysis is the availability of suitable spectral libraries for peptide identification and quantification. Several studies were performed addressing the evaluation of spectral library performance for protein identification in DIA measurements. But so far only few experiments estimate the effect of these libraries on the quantitative level.

In this work we created a gold standard spike-in sample set with known contents and ratios of proteins in a complex protein matrix that allowed a detailed comparison of DIA quantification data obtained with different spectral library approaches. We used in-house generated sample-specific spectral libraries created using varying sample preparation approaches and repeated DDA measurement. In addition, two different search engines were tested for protein identification from DDA data and subsequent library generation. In total, eight different spectral libraries were generated, and the quantification results compared with a library free method, as well as a default DDA analysis. Not only the number of identifications on peptide and protein level in the spectral libraries and the corresponding DIA analysis results was inspected, but also the number of expected and identified differentially abundant protein groups and their ratios.

We found, that while libraries of prefractionated samples were generally larger, there was no significant increase in DIA identifications compared with repetitive non-fractionated measurements. Furthermore, we show that the accuracy of the quantification is strongly dependent on the applied spectral library and whether the quantification is based on peptide or protein level. Overall, the reproducibility and accuracy of DIA quantification is superior to DDA in all applied approaches.

Data has been deposited to the ProteomeXchange repository with identifiers PXD012986, PXD012987, PXD012988 and PXD014956.

## Abstract (precis)

The impact of different approaches for spectral library generation used by DIA was analyzed on identification and quantification level using a gold standard spike-in data set. We included approaches using repetitive measurements of the original samples as well as methods applying peptide and protein pre-fractionation before library generation. The comparisons show, that prefractionation generally increases the number of identified peptides and protein groups, whereas the ground truth quantification ratios could be well approximated using libraries generated by repetitive measurements.

## Graphical Abstract

**Figure:**
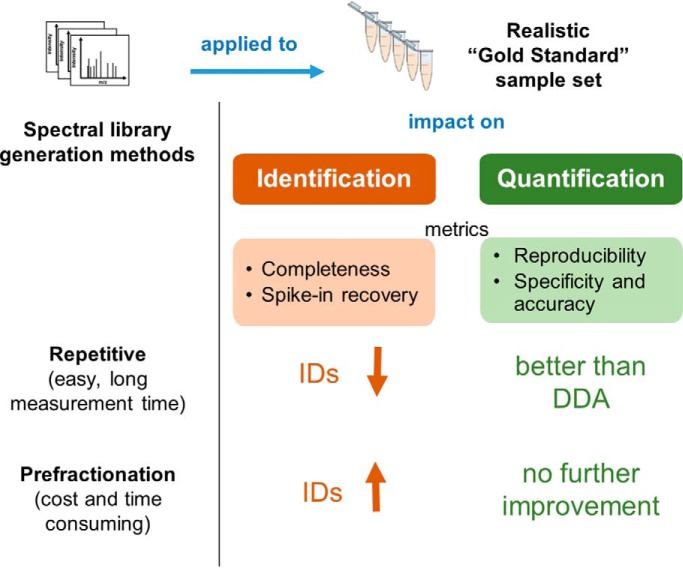

- Provision of data generated on the basis of a gold standard spike-in sample set.
- Choice of spectral library has great impact on identification and quantification.
- DIA is superior to DDA in quantification reproducibility, specificity and accuracy.
- DIA outperforms DDA in the quantification of low protein amounts.
- Quantification on peptide level is generally preferable.

Data-independent acquisition (DIA)^1^ is gaining more and more interest as a method for reliable and comprehensive label-free quantification (LFQ) of proteomics data and was already successfully applied in several clinical and biomarker discovery studies (1–4). So far, most liquid chromatography coupled tandem mass spectrometry (LC-MS/MS) approaches are relying on data-dependent acquisition (DDA) in which typically the most abundant precursor ions of an MS1 survey scan are selected for fragmentation and acquisition of respective MS2 spectra (5–7). Thus, only selected precursors can be identified leading to a loss of information, especially for low abundant precursors (8). In addition, the stochastic nature of data-dependent precursor selection results in only partially reproducible LC-MS/MS data (9, 10). At the time of writing this manuscript, quantification of DDA data is usually achieved on the basis of MS1 chromatographic peak area, which is prone to interferences, especially in complex samples (11, 12). To reliably determine relative protein abundances in a label-free experiment, targeted approaches such as selected reaction monitoring (SRM, (13)) or parallel reaction monitoring (PRM, (14)) are being used. In SRM a predefined set of up to 100 precursors is chosen for fragmentation before acquisition, resulting in highly accurate quantification even at low abundance at the cost of the depth of the analyzed proteome (5, 13). Although DDA is commonly used for discovery proteomics, SRM/PRM can be applied for *e.g.* the verification of a predetermined set of proteins.

In contrast to DDA and SRM/PRM, in which only a subset of all precursor ions present in a complex sample is fragmented and analyzed in MS2, DIA methods allow the fragmentation of all possibly generated precursor ions by cycling through predefined *m*/*z* windows along the whole survey scan range (15, 16). Although this allows acquisition of nearly complete MS2 data, the direct correlation between precursor and its fragment ions is lost resulting in the need for more complex data analysis algorithms. Typically, spectral libraries, which are generated from previous DDA measurements, are used to infer the precursor peptide - fragment connection and thus allow peptide and protein identification (17, 18). In addition, spectral library-free approaches are being developed (*e.g.* DIA-Umpire and DirectDIA), but currently these cannot identify as many precursors as the spectral library-based approaches (19, 20). DIA allows quantification on MS2 level through extraction of fragment ion chromatograms, which are less prone to interference than MS1 peak areas. This makes the quantification more accurate and reliable than in DDA (15, 21, 22).

Although DIA was first described in 2004 (23) proteomics scientists show increasing interest in it for the past few years mainly because of vast improvements in instrumentation and software. Several studies revealed the advantages of DIA against DDA methods. Most of them demonstrated an improved peptide and protein identification rate using spectral library-based DIA (*e.g.* (24, 25)) even in single shot analyses (16). In addition, a few studies showed an improved quantification reproducibility based on the coefficient of variation (CV) between technical replicates (1, 26). But more detailed analyses especially of quantification performance regarding specificity and accuracy of differential abundance detection are rarely performed because they rely on the use of suitably tailor-made samples.

Because DIA performance relies on the use of suitable spectral libraries different methods for the library generation were proposed and evaluated: Although Rosenberger *et al.* developed a generic large-scale human spectral library database (27), it was shown later on that the use of large external library repositories is inferior in terms of number of identifications (28). The in-house generation of project and sample-specific libraries benefits from the use of the same LC-MS setup as used in DIA measurements (1, 28). It is common to combine repeated DDA measurements of the same sample to maximize peptide coverage, but libraries can also be extended by sample pre-fractionation. For instance, Govaert *et al.* evaluated fractionation on protein, peptide and acquisition level and showed that all methods increased the library size, whereby protein fractionation using SDS-PAGE proved to be the most effective method (29). The quality of a spectral library often is assessed on the basis of library size (number of included precursors, peptides and proteins) and the number of yielded extractions from DIA data instead of the accuracy of the contained spectra. Just recently, methods to create libraries *in-silico* were developed (30–32). These take a suitable protein sequence database as input and computationally predict peptide fragments, which can be compiled into spectral libraries.

In this study, we exhaustively evaluated the quality of different spectral libraries regarding their identification and especially their quantification potential in DIA data analysis. Therefor we created a gold standard spike-in sample set consisting of C2C12 (immortalized mouse myoblast) cell lysate as constant background spiked with 13 proteins in five different concentrations. These standardized samples, although mimicking complex clinical samples with the proteins of interest present over a broad concentration range, allowed a detailed analysis of quantification reproducibility as well as specificity of and accuracy in differential abundance detection. The five samples were measured by DDA and DIA in triplicate, whereby the following approaches for spectral library usage were realized for DIA data analysis: On the one hand we used repeated DDA runs of in-solution digests and on the other hand prefractionation of samples on protein and peptide level to generate a range of different spectral libraries in Spectronaut 11 (SN, Biognosys AG, Schlieren, Switzerland) using the identification results of Proteome Discoverer 2.2 (PD, Thermo Fisher Scientific, Dreieich, Germany). In addition, we used Spectronaut Pulsar's protein identification algorithm for spectral library generation of two representative DDA data sets. These spectral library-using approaches were compared with the spectral library-free DirectDIA analysis by SN Pulsar, which has not been thoroughly described in literature so far, and an analysis of DDA data using OpenMS (33, 34). The generated data were thoroughly evaluated regarding peptide and protein identification and especially regarding quantification performance. In addition to CVs, we determined the significance of the induced spike-in abundancy changes and their correlation with the theoretical values. Furthermore, we analyzed the effectiveness to identify proteins that were differentially abundant (true positives) and the analysis specificity in terms of false positive rate. The focus of the presented analyses was the evaluation of the impact of differently generated spectral libraries on one DIA data set, not to benchmark different MS technologies or tools on the same sample respectively data set, as was for example done in (35). In the latter Navarro *et al.* benchmarked the quantitative performance of different mass spectrometers and analysis tools but used the same libraries for DIA extraction. The main part of this work was performed using data generated by Spectronaut. Nevertheless, we additionally analyzed the data set with OpenSWATH (36) and will highlight some of the results (for detailed results, see supplemental document).

## Experimental Procedures

### Sample Preparation

Frozen C2C12 cells were warmed up within a water bath (37 °C) and taken into culture in a 58 cm^2^ Petri dish (Sarstedt, Nümbrecht, Germany) within 10 ml DMEM (Gibco®, Thermo Fisher) standard medium containing 15% (v/v) FCS (Gibco®), 2% (v/v) sodium pyruvate (Biochrom, Berlin, Germany), 1% (v/v) non-essential amino acids (Biochrom) and 1% (v/v) penicillin/streptomycin (Pan Biotech, Aidenbach, Germany). The cells were cultivated in an incubator (37 °C, 5% CO_2_), the medium was changed every 2 days and the cells were split at a confluency of approx. 70%. For this, the cells were washed with 5 ml PBS (Gibco®), then detached with 1.5 ml 0.05% Trypsin/1 M EDTA (Gibco®) for 3 min inside the incubator and further the activity of trypsin was stopped by the addition of standard medium.

Before lysis, cells were pelleted by centrifugation at 16,000 × *g* for 10 min and then lysed in 30 mm TrisHCl, pH 8.5, 7 m urea and 2 m thiourea using glass beads and sonication (4 × 1 min on ice). After lysate transfer into a fresh tube, glass beads were washed with distilled water, the resulting solution was combined with the lysate (resulting in 5.3 m urea and 1.5 m thiourea concentrations) and cleared by centrifugation at 16,000 × *g* for 10 min.

As spike-in proteins we chose 13 non-mouse proteins with varying physico-chemical properties (see supplemental Table S1 for detailed information and UniProt accessions): human α-synuclein, β-lactoglobulin from bovine milk, fibrinogen α, β and γ from human plasma (Merck Millipore, Darmstadt, Germany), glucose oxidase from *Aspergillus niger*, human hemoglobin α and β, lipases 1, 2 and 3 from *Candida rugosa*, lysozyme from chicken egg white and myoglobin from equine skeletal muscle (Sigma-Aldrich, part of Merck KGaA, Darmstadt, Germany, unless otherwise stated). The fibrinogen α, β and γ were contained in the same solution, as were the hemoglobin α and β and the lipases 1, 2 and 3 respectively. Therefore, the relative amounts of these proteins are equal in all spike-in levels. The spike-ins were combined in a manner to yield in a comparable overall sample amount and physiologically plausible protein concentrations.

The gold standard spike-in sample set (GS) consisting of five samples was prepared as follows: a constant amount of C2C12 lysate as background matrix was spiked with varying amounts of the 13 spike-in proteins in 50 mm ammonium bicarbonate (AmBic) as specified in Table I.

**Table I:** Composition of the gold standard spike-in sample set consisting of a constant C2C12 cell lysate background and varying amounts of 13 non-mouse spike-in proteins. Shown are the pmol amounts of the spike-in proteins for each of the five sample states

	UniProt accession	Amount of spike-in proteins (pmol)				
		Sample 1	Sample 2	Sample 3	Sample 4	Sample 5
α-synuclein (pmol)	P37840	1	10	0.5	0.1	5
β-lactoglobulin (pmol)	P02754	0.5	0.1	5	10	1
Fibrinogen (pmol)	P02671, P02675, P02679	10	5	1	0.5	0.1
α, β, γ each						
Glucose oxidase (pmol)	P13006	0.1	1	10	5	0.5
Hemoglobin (pmol)	P69905, P68871	0.5	5	10	1	0.1
α, β each						
Lipase (pmol)	P20261, P32946, P32947	0.1	0.5	1	5	10
1, 2, 3 each						
Lysozyme (pmol)	P00698	5	10	0.1	0.5	1
Myoglobin (pmol)	P68082	1	0.1	5	10	0.5
C2C12 lysate (μg)		20	20	20	20	20

For spectral library generation (in-solution digest, protein fractionation and peptide fractionation) C2C12 lysate was mixed with equal amounts of the spike-in proteins (called master mix, MM, in the following) as specified in supplemental Table S2.

#### 

##### In-solution Tryptic Digestion

GS samples, MM for the in-solution and peptide fractionation library (sample composition as specified in Table I and supplemental Table S2) were prepared as follows.

After reduction with dithiothreitol (DTT, final concentration of 5 mm) for 20 min at 56 °C proteins were alkylated with iodoacetamide (13.75 mm final concentration) at ambient temperature for 30 min in the dark. Samples were diluted with 50 mm AmBic to an urea concentration <1.5 M and digestion was carried out using trypsin (Serva, Heidelberg, Germany) at an enzyme to substrate ratio of approx. 1:27 at 37 °C overnight. The digestion was stopped by adding trifluoroacetic acid (TFA) to a final concentration of 0.5%. After centrifugation the supernatant was collected, and the peptide concentration was determined by in-house amino acid analysis (AAA) (37). Before LC-MS/MS analysis was performed, the iRT kit provided by Biognosys, which is required for DIA analysis using Biognosys' SN, was added according to the manufacturer's instructions. In brief, solubilized iRT peptides were diluted 1:10 in 0.1% TFA and 1 μl was added to each sample.

##### Protein Fractionation by SDS-PAGE and In-gel Tryptic Digestion

For protein fractionation by SDS-PAGE the MM sample (composition as specified in supplemental Table S2) was reduced and alkylated as described in In-solution Tryptic Digestion. Per lane, 40 μg were loaded onto an Invitrogen™ Bolt™ 4–12% Bis-Tris Plus Gel (Thermo Fisher Scientific) and proteins were separated at 200 V in MOPS buffer. After staining with SimplyBlue SafeStain (Thermo Fisher Scientific) each lane was cut into 10 slices, which were destained before subjection to overnight trypsin digestion using 0,12 μg trypsin per gel slice. Peptides were eluted twice using 0.1% TFA: acetonitrile (ACN) 1:1. The supernatants of each fraction were combined, vacuum dried and resuspended in 0.1% TFA 10 different fractions. Before LC-MS/MS analysis, 0.5 μl of solubilized iRT peptides were added to each sample.

##### Peptide Fractionation by High pH Reversed Phase Chromatography

Peptide fractionation was done using the “Pierce High pH Reversed-Phase Peptide Fractionation Kit” (Thermo Fisher Scientific) according to the manufacturer's manual. In brief, the peptide sample prepared as described in In-solution Tryptic Digestion was vacuum-dried, resuspended in 300 μl 0.1% TFA and loaded onto the equilibrated column. Peptides were eluted stepwise with increasing ACN concentration in 0.1% triethylamine into 8 fractions, that were vacuum-dried and resuspended in 0.1% TFA. Finally, peptide concentrations were determined by in-house AAA. Before LC-MS/MS analysis, 0.5 μl of solubilized iRT peptides were added to each sample.

### Mass Spectrometric Acquisition

All samples (library samples, DDA and DIA analysis samples) were analyzed using the following LC-MS/MS setup: For LC separation the nanoHPLC system Ultimate 3000 (Thermo Fisher Scientific) was used with a PepMap 100 C18 (100 μm ID × 2 cm, particle size 5 μm, pore size 100 Å; Thermo Fisher Scientific) as precolumn and a PepMap C18 (75 μm × 50 cm, particle size 2 μm, pore size 100 Å; Thermo Fisher Scientific) as analytical column. Peptides were separated by a 120 min gradient using 0.1% formic acid (FA) as buffer A and 84% ACN in 0.1% FA as buffer B. The gradient was run from 5% to 40% buffer B. Subsequently, peptides were ionized by electrospray ionization and transferred into a Q Exactive HF mass spectrometer (Thermo Fisher Scientific). The capillary temperature was set to 250 °C and the spray voltage to 1600 V. The lock mass polydimethylcyclosiloxane (445.120 m/z) was used for internal recalibration.

For DDA MS runs (library generation and DDA analyses), the mass range of MS1 full scans was set to 350–1400 *m*/*z* with a resolution of 60,000 at 200 *m*/*z* (AGC 3 × 10^6^, 80 ms maximum injection time). HCD fragmentation of the Top10 abundant precursor ions was performed at 27% NCE. The fragment analysis (MS2) was performed with a resolution of 30,000 at 200 *m*/*z* (AGC 1 × 10^6^, 120 ms maximum injection time, 2.2 *m*/*z* isolation window).

For DIA MS runs, the MS1 full scans were performed at a mass range of 350–1400 *m*/*z* with a resolution of 120,000 at 200 *m*/*z* (AGC 3 × 10^6^, 20 ms maximum injection time). Fragment analysis (MS2) was subdivided into 22 DIA isolation windows of equal width (49 *m*/*z* wide) using a resolution of 30,000 at 200 *m*/*z* (AGC 3 × 10^6^, auto maximum injection time). Stepped collision energy was used (25.5%, 27 and 30% NCE).

Sample analyses, containing the spike-in proteins, in DDA and DIA mode were performed with 200 ng sample peptide amount (corresponding to ∼1 to 100 fmol spike-ins), whereas for library generation up to 800 ng of peptides per sample were injected.

The raw spectrometric data and the spectral libraries have been deposited to the ProteomeXchange Consortium (http://proteomecentral.proteomexchange.org) via the PRIDE partner repository (38) with the data set identifiers PXD012986, PXD012987, and PXD012988.

#### 

##### Spectral Library Generation

DDA data were searched with PD using Mascot 2.5 (Matrix Science Ltd, London, UK, (39)) as peptide search engine and Percolator (40) for the refinement of target-decoy estimation.

For all searches, DIA and DDA, the following common settings were used- as fixed modification, only aarbamidomethylation at C was set, whereas as variable modifications oxidation (M), Gln->pyro-Glu (N-terminal Q), deamidated (NQ), ammonium (DE) and ammonia-loss (N, N-terminal C) were allowed because of sample preparation

- A maximum of two missed cleavages was allowed.
- The precursor tolerance was set to 5 ppm and the fragment tolerance to 20 mmu.
- The cleavage enzyme Trypsin (cleavage at each K and R, unless followed by P) was used.

For DDA identifications, a database consisting of the UniProt reference mouse proteome (release 2017_12, 52548 protein entries), the cRAP contaminants (unchanged since January 2015, 115 entries), the iRT protein for DIA retention time calibration and a database of the spike-in proteins was used. Additionally, to the 13 spiked-in proteins several proteins that were identified together with them in prior experiments (data not shown) were added to the database, containing altogether 160 protein accessions in the spike-in database (compare Statistical Analysis). Thus, the complete protein database used in this study contained 52,824 entries. The searches to create the spectral library were refined using Percolator, allowing only high confidence peptides in PD. Spectral libraries were generated using the spectral library generation function of SN using mostly the default settings. In brief, PD confidence level “high” was used for identification with SN protein inference enabled. Fragment ions between 300–1800 *m*/*z*, with minimum 3 amino acid length and minimum relative intensity of 5 were considered. Precursors with less than 3 fragment ions were removed. For the generation of SN Pulsar spectral libraries, DDA raw data were directly loaded into SN Pulsar and spectral library generation was done using the same settings as described for SN 11. An overview on all spectral libraries used in this work regarding library creation can be found in Fig. 2 and Table II (supplemental Table S3 shows some additional information like preparation time), and regarding library content (their constituting precursors, peptides and protein groups) in supplemental Table S5. In total, eight different libraries are described here; whereby the most complex spectral library “IS and F all 78” was created from DDA data of the other 5 non-Pulsar libraries and 6 additional measurements of the “MM IS 6” sample.

**Table II:** Overview of analyzed DIA libraries. The names of each library consists of the spike-in types (MM: master mix, i.e. mixture of background and spike-ins in fixed amount, GS: gold standard, i.e. the same samples as measured by DIA), the digest type (IS: in-solution, F: fragmented and therefore in-gel), whether fractionation was performed and on which level (Prot: protein fraction, Pep: peptide fractionation), finally, the number represents the number of MS runs for the library. The libraries generated by Pulsar instead of ProteomeDiscoverer are indicated by the respective prefix

Name	Spike-in type	Digest type	Fractionation	No of runs	Software
MM IS 6	Constant	In-solution	No	6	PD+SN11
GS IS 15	Varying	In-solution	No	15	PD+SN11
GS IS 30	Varying	In-solution	No	30	PD+SN11
MM F Prot 20	Constant	In-gel	Protein	20	PD+SN11
MM F Pep 16	Constant	In-solution	Peptide	16	PD+SN11
IS and F all 78	Combined	Combined	Combined	78	PD+SN11
Pulsar GS IS 15	Varying	In-solution	No	15	SN Pulsar
Pulsar MM F Prot 20	Constant	In-gel	Protein	20	SN Pulsar

##### DDA Data Analysis Using OpenMS

DDA data were analyzed with a KNIME (41) workflow using OpenMS and PIA (42, 43) nodes (workflows are deposited inside PRIDE with the data). In brief, MS/MS spectra were first converted into centroided mzML using the vendor algorithm of msConvert (ProteoWizard version 3.0.10112 (44)). Afterward, they were identified using the target-decoy approach with Mascot 2.5, MS-GF+ (45) and X!Tandem (46) and searches were combined using PIA, maintaining a peptide spectrum match and protein level false discovery rate of 1%. Peptide-features were detected and quantified using the FeatureFinderMultiplex. This algorithm uses the areas under the curves of the peptide isotope ion trails in the MS1 scans for the respective quantifications. The identifications were mapped to the features, aligned and normalized using the respective OpenMS nodes. Before the protein quantification using Top3 peptide abundancies, protein inference was conducted using PIA. The quantities for purely sequence based peptides was inferred from the quantities of peptides distinguishing different modifications and charge states by summing up the respective raw quantities, which is the default approach in OpenMS. The resulting peptide and protein quantifications were statistically analyzed as described below (see Statistical Analysis).

##### DIA Data Analysis Using Spectronaut 11 and Pulsar

DIA data were analyzed with SN 11 using the following settings. Calibration was set to non-linear iRT calibration with precision iRT enabled. Identification was performed using 1% q-value cutoff on precursor and protein level whereas the maximum number of decoys was set to a fraction of 0.5 of library size. The mass tolerance for matching precursor and fragment ions was set to dynamic (default), which lets SN determine the optimal value. For quantification interference correction was enabled with at least three fragment ions used per peptide, major and minor group quantities were set to mean peptide and mean precursor quantity, respectively with Top3 group selection each. Quantity was determined on MS2 level using area of XIC peaks with enabled cross run normalization. A complete description of all parameters can be found in supplemental Table S4.

In addition, DirectDIA was performed on the DIA data set with SN Pulsar using the same settings as described above.

##### Statistical Analysis

The quantitative data were exported from SN using an export schema, which allowed a statistical analysis for peptide and protein group relative abundancies. In the following we used all quantities, which had a valid value for at least one run, which is analogous to using the “sparse” setting in SN, unless stated otherwise. All data from the DIA and DDA measurements were analyzed by the same workflow using KNIME and R. The respective workflow can be found in the ProteomeXchange upload. On both levels, peptide and protein, the following analyses were conducted: first, missing values were imputed to a value of 0. The data were transformed using the inverse hyperbolic sine function (arcsinh), which has similar characteristics as the logarithm in the given numeric range but is defined for 0. Afterward, an analysis of variance (ANOVA) model was fitted to the transformed data. As a post-hoc test Tukey's honest significance test was conducted, to determine, which spike-in states were significantly differential. Finally, the ANOVA *p* values were corrected for multiple testing using the Benjamini-Hochberg procedure. To calculate the fold changes and log2 ratios between the spike-in states, the average quantities between the replicates for each state were calculated, leaving out the missing values instead of trying to impute them.

To account for impurities introduced by the spike-in protein solutions additionally to the 13 spike-in proteins 147 protein sequences were added to the protein database used for identifications. These proteins were identified by prior MS analyses of the spike-in protein solutions (data not shown), which were not highly purified. These “spike-in contaminants” are not expected to be found in all spike-in samples because of their low amount, but are nevertheless added to the protein database to allow an identification of the respective spectra. Thus, they are neither considered true positives (TP) nor false positives (FP) in the remaining analyses.

Besides these spike-in contaminants, there might be more peptides in the spike-in solutions, which could be detected and mapped to mouse proteins. To account for these FP, the abundancies of proteins, which are detected to be differential, were correlated to the known spike-in abundancies, using Pearson's correlation coefficient. The data were filtered if the correlation was higher than 0.9. Additionally, a fold change (FC) filter was applied, filtering out all candidates with relatively small FCs (FC < 1.3).

In all analyses, unless stated otherwise, the TP are the spike-in proteins, which were correctly identified to be differentially abundant. FP are detected as differential but are none of the spike-in proteins. To calculate the FP rate of the differential proteins, the number of differential FPs was divided by all differential proteins, *i.e.* the number of TP, the spike-in contaminants (which are regarded as TP in this calculation, as they obviously are regulated) and the FP.

The accuracy of differential abundance detection was assessed based on the mean absolute percentage error (MAPE), which is a percental measure for the deviation of all spike-in ratios from the theoretical ones. The formula for the calculation is:

$$MAPE=\frac{1}{n}\sum_{t=1}^{n}\left|\frac{A_{t}-F_{t}}{A_{t}}\right|$$

where *n* is the number of possible abundance ratios (ten ratios for the five different states), *A_t_* is the theoretical log2 value of the abundance ratio and *F_t_* the median of the measured log2 ratios for the respective states. This allows to compare the accuracy of the quantifications using one objective value.

To compare the SN results with results generated by another tool, we performed an analysis using OpenSWATH. The analysis followed the basic tutorial settings of OpenSWATH using Mascot search results, iProphet (47) for the combination of results, Mayu (48) for the FDR estimation and spectraST (49) for the actual library generation. The DIA data was analyzed by OpenSWATH and PyProphet (50) and the quantified features were aligned using TRIC (51). For more information on the commands and parameters, see the supplementary file. The same KNIME workflow as for the SN and OpenMS data was used for the final analyses.

### Experimental Design and Statistical Rationale

As described in the prior paragraphs a total of 78 LC-MS/MS runs were analyzed for the spectral library generation (compare supplemental Table S3, the number in the library names reflects the number of MS runs used for the creation of the library, the “MM IS 6” were additionally replicated with 800 ng and analyzed with the “IS and F all 78” library). For the DDA and DIA analysis, the described five spike-in states were measured in triplicates to reflect a common replicate number, resulting in 15 LC-MS/MS runs per method. For the retention time alignment of the DIA analyses, the iRT kit provided by Biognosis was applied, as described in Sample Preparation. MS1 and MS2 spectra were acquired for the DDA and DIA analyses as described in Mass Spectrometric Acquisition.

## Results

**Fig. 1.**
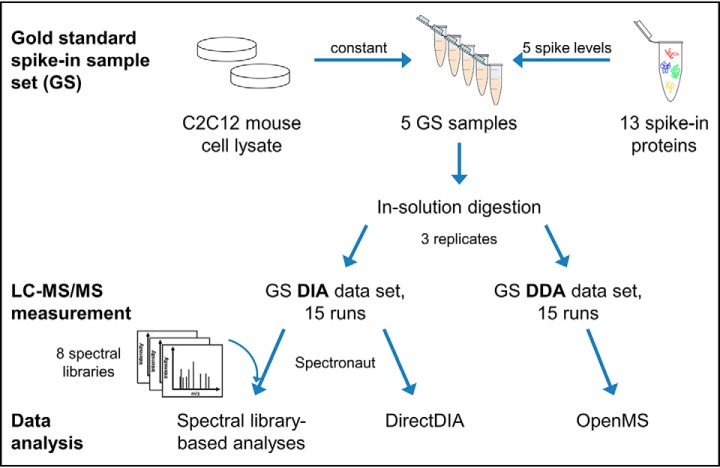
**Study design for setup and analysis of the gold standard spike-in sample set (GS).** The GS was created by spiking 13 proteins into C2C12 cell lysate with 5 defined concentration levels. After in-solution trypsin digestion, each sample was subjected to triplicate LC-MS in DIA and DDA mode. Although GS DIA data were either analyzed in a spectral library-based or a spectral library-free (DirectDIA) approach using Spectronaut, GS DDA data were analyzed using OpenMS for identification and quantification.

To thoroughly analyze the performance of different label-free quantification approaches, we generated a gold standard spike-in sample set (GS) consisting of C2C12 cell lysate as constant background and 13 non-mouse proteins each in five different concentrations (study design see Fig. 1). By using spike-in proteins with varying physico-chemical properties, we were able to mimic a complex biological sample containing the proteins of interest—the proteins to be relatively quantified—over a broad concentration range. The individual spike-ins were combined in a way to keep the overall protein concentration constant between the five samples to facilitate good comparability—a prerequisite for successful normalization for relative quantification. We used this sample set to compare identification and quantification performance, on the one hand between DDA, DirectDIA, and spectral library-based DIA and on the other hand we evaluated the influence of different spectral libraries. Because the actual differentially abundant proteins (true positives, TPs)—the spiked-in non-mouse proteins—can clearly be distinguished from false positive (FP) background proteins, the quantification specificity can be evaluated in detail. Regarding relative quantification, the GS enabled us to compare the protein differential abundance ratios obtained by the various analyses with the theoretic values to estimate quantification accuracy.

### Generation of the Gold Standard Spike-in Sample Set

For the creation of a sample set suited for the verification of quantification reproducibility, specificity and accuracy, we used whole mouse cell lysate as constant background, representing a complex biological sample, complemented with 13 purified non-mouse proteins in varying concentrations. In summary, six human, three animal and four fungal proteins with varying characteristics in terms of length, hydrophobicity and isoelectric point (pI) as well as with minimal tryptic peptide overlap with the C2C12 cell lysate were selected. Proteins consisting of more than one subunit (hemoglobin and fibrinogen) and the fungal lipases were present in sample mixtures, whereas all other proteins were present in separate solutions (see Table I and supplemental Table S1 for sample composition and spike-in protein details, respectively). Before preparation of the GS, each protein sample was characterized individually by LC-MS/MS verifying the presence of the 13 spike-in proteins. Beside these, 147 additional proteins were identified (in the following termed “spike-in contaminants,” see supplemental FASTA file containing the spike-in protein sequences and the spike-in contaminants). The thirteen proteins result theoretically in 948 possible tryptic peptides (considering amino acid sequence lengths ranging from 6 to 50 and allowing up to two missed cleavages) ensuring enough statistical power for data analysis.

Altogether, the GS consisted of five different samples that contained the same amount of C2C12 lysate but varying concentrations of each spike-in protein. The individual spike-in amounts were shuffled to keep the overall protein concentration constant (see Table I). We used five physiologically plausible spike-in amounts of 1.25 to 125 nm per spike-in protein (as deduced from plasma protein concentration ranges (52)) in a 0.27 μg/μl overall protein concentration, generating a broad range of protein ratios between 2 and 100. For LC-MS/MS analysis of the GS, each sample was measured in triplicate in DDA and DIA mode using the same instrumental setup (nano-LC coupled to Q-Exactive HF).

### Spectral Libraries

**Fig. 2.**
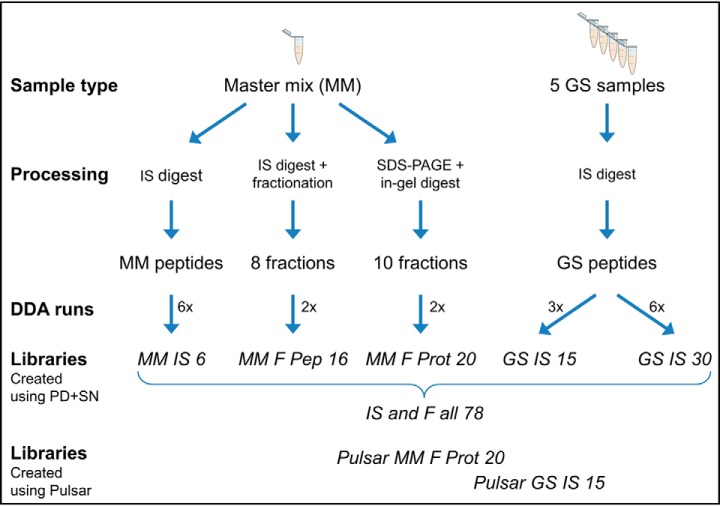
**Overview on the eight used spectral libraries.** For spectral library generation we used the in-solution (IS) digested GS samples or a master mix (MM) sample. Either an MM IS digest was directly subjected to LC-MS measurement in DDA mode or it was fractionated (F) on protein (Prot) or peptide (Pep) level. Spectral libraries were created from DDA data of the five different sample and processing types using a combined PD+SN approach. All DDA measurements were combined into a comprehensive “IS and F all 78” library. In addition, two libraries were also created using SN Pulsar. (Abbreviations: MM master mix, IS in-solution, F fractionation, Pep peptide, Prot protein, GS gold standard).

For DIA data analysis of the GS, eight different spectral libraries were generated using (1) two different sample types, (2) varying sample preparation methods, and (3) two different search engines (see Fig. 2 for an overview). In addition to the samples of the GS, we used a protein master mix (MM) containing the spike-in proteins in equal amounts within the C2C12 matrix (1), thus ensuring all proteins of interest to be present for successful identification. Although the GS was used for in-solution (IS) digest followed by mass spectrometric acquisition only, the MM sample was also subjected to fractionation (F) on protein (Prot) or on peptide (Pep) level to create more complex libraries (2). For spectral library generation the individual IS and F samples were analyzed by LC-MS/MS in DDA mode. As the most comprehensive library, a combined library containing all DDA runs was generated (1+2). All the above described libraries were created using Proteome Discoverer (PD) identification with Mascot as search engine and Spectronaut (SN) for spectral library creation. To examine the influence of the search engine, two representative libraries were also generated using SN Pulsar (3).

**Fig. 3.**
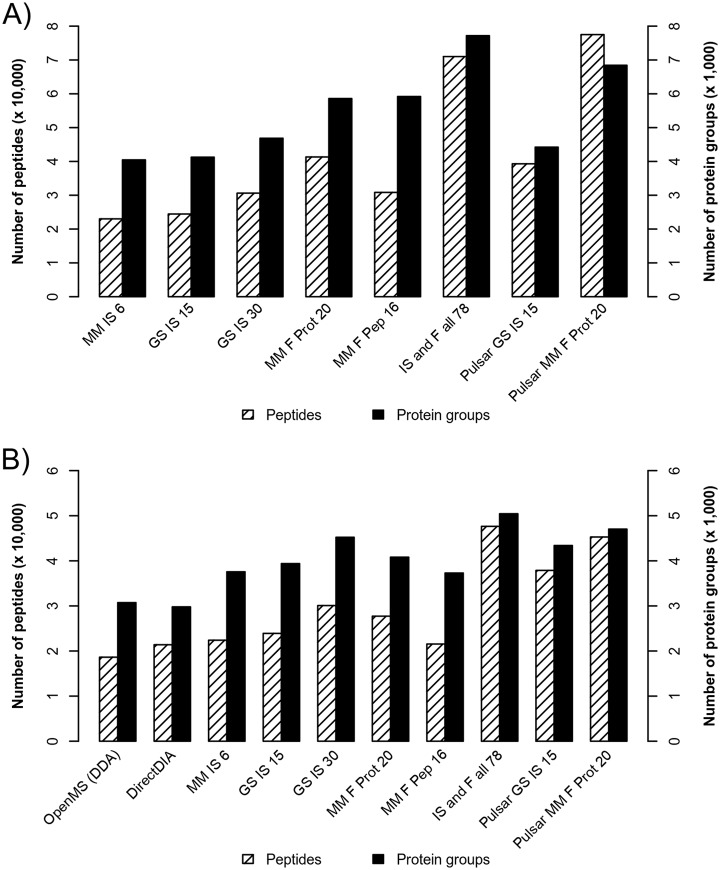
***A*, Size of the generated spectral libraries.** Shown are the number of peptides and protein groups included in each spectral library. The smallest spectral library based on DDA measurements is the “MM IS 6” library. The highest number of peptides are included in the “Pulsar MM F Prot 20” library whereas the largest library in terms of protein groups is the “IS and F all 78.” *B*, Identifications obtained from triplicate measurements of the five spike-in samples. Given are the accumulated numbers of identified peptides and protein groups from the spiked C2C12 cell lysate samples for the DDA analysis using OpenMS and the DIA analyses performed in SN. Although DDA extracted the lowest number of peptides and DirectDIA the lowest number of protein groups, the highest number of identifications was achieved with the “IS and F all 78” spectral library based DIA analysis.

The simplest spectral library generated with six DDA runs of IS digested MM sample (“MM IS 6”) contained the least number of peptides and protein groups (23,020 peptides and 4046 protein groups, see Fig. 3, for the complete numbers see supplemental Table S5). This MM sample mimics a sample pool, which can be generated if the amount of each individual sample is too low for multiple MS analyses. We additionally created the “MM IS 6” library with 4-fold increased peptide concentration, but this resulted only in a marginal increase in library size (data not shown). In contrast, increasing the number of MS runs from 15 in the “GS IS 15” library (24,457 peptides and 4,126 protein groups) to 30 (“GS IS 30”) resulted in a 25 and 13% larger library on peptide and protein level, respectively. Sample fractionation is often performed as a strategy to increase the spectral library depth (29). We performed fractionation on protein and peptide level (“MM F Prot 20” and “MM F Pep 16”), which further increased the spectral library size. Here protein fractionation yielded in higher number of peptides than fractionation on peptide level (41,322 peptides and 5859 protein groups in “MM F Prot 20” and 30,838 peptides and 5919 protein groups in “MM F Pep 16”). We observed an improvement of library size of up to 39% on peptide level by measuring each fraction twice instead of just once (data for single measurements not shown). As expected, combining all sample runs into one spectral library (“IS and F all 78”) resulted in the largest library with 7718 protein groups and 71,008 peptides. We also observed an impact of the used spectrum search engine on library size. SN Pulsar, although using the same raw data, was able to extract considerably more peptides and proteins than the respective combined PD and SN approach (see Fig. 3 and supplemental Table S5 for actual numbers).

### Peptide and Protein Identification

To evaluate the performance of DIA for the analysis of the GS data set, we used different DIA approaches including spectral library-free “DirectDIA” and spectral library-based analyses using the different spectral libraries described above. These were compared with a standard DDA approach using OpenMS examining ten analyses in total. Initially, we assessed the number of identified peptides and protein groups. Overall, spectral library-based analyses resulted in higher numbers of identified protein groups compared with the DDA- and DirectDIA-based analyses. The latter led to the lowest number of identified protein groups with 2979 over all 15 analyzed samples (see Fig. 3*B*, for the actual numbers see supplemental Table S6). Among the spectral library-based analyses a library size dependent increase in the number of identified proteins and peptides was observed for the DIA analysis using libraries generated with the combined PD and SN approach from IS samples. In brief, the simplest IS library “MM IS 6” library extracted 3757 proteins and 22,397 peptides, whereas the “GS IS 15” library extracted 3938 proteins and 23,911 peptides. Including additional DDA runs within spectral library generation, as realized in the “GS IS 30” library, resulted in 4522 proteins and 30,086 peptides. As the results using the “MM IS 6” and the “GS IS 15” libraries are comparable, using a sample pool for library generation seems to be a suitable approach for DIA data analysis if *e.g.* only small amounts of sample are available. Surprisingly, although the fractionation libraries (“MM F Prot20” and “MM F Pep 16”) were larger than the IS libraries (“MM IS 6,” “GS IS 15,” and “GS IS 30”), lower peptide and protein group identifications were observed (compare numbers in supplemental Table S5 and S6). The highest number of peptides and protein groups was identified with the most complex library “IS and F all 78” (47,646 peptides and 5044 protein groups). In terms of identifications the use of Pulsar instead of PD+SN for spectral library generation proved to be beneficial, *e.g.* 63% more peptides and 15% more protein groups were extracted with the library “Pulsar MM F Prot 20” than with “MM F Prot 20.” This must be cautioned though: even as it is possible to set the FDR filters in Pulsar, the analysis is rather a black box. The actual decoys and FDR calculations cannot be inspected, at least when using the Spectronaut implementation of Pulsar.

#### 

##### Library Recovery

Investigating the library recovery in more detail - which is defined as the number of peptides or protein groups actually identified from the GS data set compared with the respective library content - we observed a very high library recovery when using the IS libraries (over 95% peptide and 89% protein recovery, see supplemental Fig. S1*A* +S1*B*). In contrast, significantly fewer peptides and proteins present in the fractionation libraries were identified in the GS data set (about 58–70%). This indicates that the IS libraries might not cover all measurable peptides from the GS sample. The fractionation libraries, especially “IS and F all 78” and “Pulsar MM F Prot 20,” on the other hand included many unrecoverable peptide spectra.

##### Completeness

For DDA it is known that when measuring comprehensive sample sets, e.g. from a clinical study, many peptides are not recorded for all samples (so called missing values (9)). Therefore, we evaluated the portion of consistently identified peptides and proteins, termed “completeness,” within each analysis and compared those among all approaches (see supplemental Fig. S1*C*). With DDA only 52% of the peptides and 65% of the protein groups were identified in all samples, whereas the DirectDIA approach provided 91% peptide and almost 99% protein completeness. In spectral library-based DIA analyses 82% to 91% of identified proteins were present in all 15 samples regardless which spectral library was used. However, on peptide level the completeness was considerably affected by the library choice. Among the PD+SN libraries the IS libraries yielded 80% to 83% completeness whereas in case of the fractionation libraries about 63% completeness was achieved. Although the peptide completeness in percent was quite low when using the most complex “IS and F all 78” library (56%), the actual number of consistently identified peptides was higher than with any other analysis. A similar effect was observed with the Pulsar spectral libraries. When comparing the Pulsar with the respective PD+SN libraries the peptide completeness dropped by about 10% whereas the Pulsar generated libraries yielded about 6700 more consistently identified peptides. As expected, peptides found in only one sample out of the 15 were mostly low abundant ones (data not shown).

In many analysis strategies, proteins or peptides must be present in at least a certain percentage of the analyzed runs to not be rejected because of too many missing values. Therefore, in supplemental Table S8 we show the percentage of protein groups and peptides, which were found in all and at least 80%, 66%, and 50% of the runs. The data shows, that the percentages on the peptide level increase with decreasing required completeness but maintains the same trend. On protein level though, requiring an identification in only 80% of the runs already results in a completeness above 90% in all analyses, except for the DDA.

**Fig. 4.**
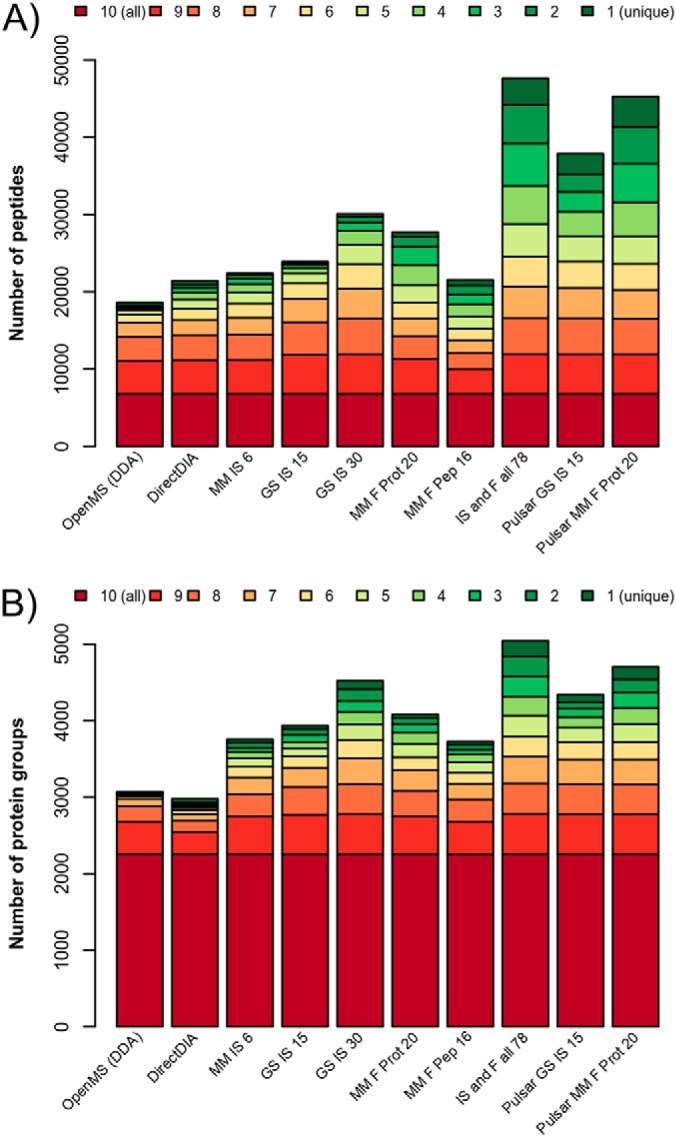
**Peptide (*A*) and protein (*B*) overlap between the different analyses.** It is shown how many peptides or proteins were found in all 10 analyses (red) or in a subset (9–2 analyses). Unique identifications are indicated by ”1” (green). The DIA analyses using libraries “Pulsar GS IS 15”, “Pulsar MM F Prot 20” and “IS and F all 78” resulted in the highest number of peptides and proteins that were not identified with any other analysis.

Besides the examination of peptide and protein completeness within each individual analysis, we further inspected the overlap of identified peptides and proteins between the ten different analyses (see Fig. 4). In total 6787 peptides and 2258 proteins were identified by all methods, whereas 3976 peptides and 205 proteins were identified in one analysis only - in the following termed unique identifications (see supplemental Table S6). The highest number of unique peptide identifications were achieved using the “IS and F all 78” (3,431 peptides) and the two Pulsar libraries (“Pulsar MM F Prot 20” 3976 and “Pulsar GS IS 15” 2652 peptides). These spectral libraries also identified the most unique proteins together with “GS IS 30”. The abundance of the uniquely identified peptides and proteins was significantly lower than the abundance of all identified peptides and proteins (see supplemental Fig. S2 for abundance box plots of those analyses with the most unique identifications). This indicates that with DIA analyses using comprehensive spectral libraries (“GS IS 30,” “IS and F all 78,” “Pulsar GS IS 15,” and “Pulsar MM F Prot 20”) detection of low abundant proteins was improved.

To evaluate the identification of the 13 spike-in proteins we examined the number of runs in which the respective protein was identified (supplemental Table S7). In total, all spike-in proteins were identified in each analysis. Individual proteins (three lipases, alpha-synuclein and myoglobin) could not be identified consistently in each run, except for the DirectDIA analysis, in which all proteins were identified throughout. This is because the DirectDIA algorithm matches and inspects all runs simultaneously for the identification of peptides and thus generally has a higher completeness than the other analyses. As expected, an abundancy-dependent identification pattern was observed. Alpha-synuclein was congruently not identified in the lowest spike-in concentration (0.1 pmol), except with DirectDIA, “GS IS 30” and DDA. The most heterogeneous identification pattern was observed for Lipase 2, which was identified in all 15 runs when using DirectDIA, but not consistently in the other analyses.

In summary, for peptide and protein identification we conclude that with spectral-library based analyses more identifications were achieved than with DDA or DirectDIA, even with the simplest library

- The larger the spectral library, the more peptide and protein identifications.
- Using SN Pulsar identifies yields in higher peptide and protein identification numbers.
- Pooling of samples for spectral library generation is a good alternative to the measurement of each individual sample.

### Peptide and Protein Quantification

The focus of this work was to compare quantification performance among the different analyses described in 3.2 and 3.3 in terms of reproducibility, specificity and accuracy.

#### 

##### Reproducibility

**Fig. 5.**
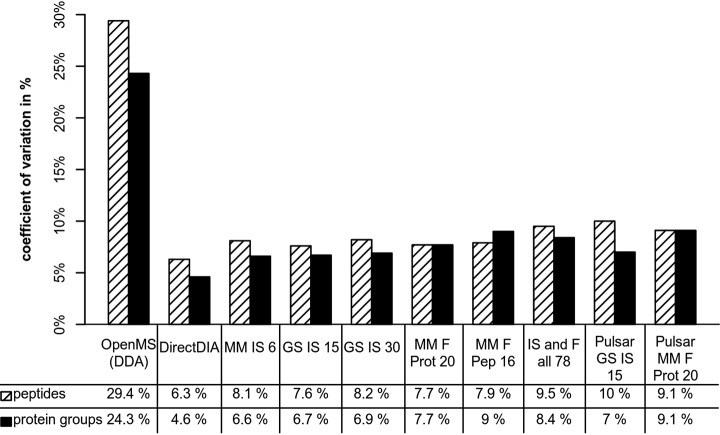
**Shown are the global median peptide and protein group CVs of the various analyses in percent.** Although the DDA analysis showed the significantly highest CV values of 29% for peptides and 24% for protein groups, the DIA CVs ranged between 6 and 10% on peptide and 5 and 9% on protein level.

First, we evaluated the reproducibility between replicates based on global coefficient of variation (CV) on peptide and protein group level (see Fig. 5 and supplemental Table S9). For the calculation of the CVs, the missing values were not imputed but simply left out. Imputing the values to 0 yields in much higher CVs and imputing to mean would lead to lower CVs (data not shown), whereas both alternatives would not reflect the actual data. In agreement with prior publications (1, 26) we found DDA showing much higher median CVs (29% on peptide and 24% on protein level) compared with DIA which ranged from 6% for DirectDIA to 10% for the “Pulsar GS IS 15” on peptide level, whereas the CVs on protein level were even lower (between 5 and 9%). In general, we observed a trend toward increasing CVs with increasing library size, which might be a result of detecting more low abundant species that naturally entail a higher variation. This was also true in case of the Pulsar libraries. Both contained more peptides and proteins than the respective libraries generated using PD+SN. An exception was the analysis using the simplest library “MM IS 6” that, although exhibiting the lowest protein CV (7%) among the spectral library-based analyses, resulted in mediocre peptide CV (8%). The difference between the PD+SN and Pulsar identification was particularly evident in case of the “GS IS 15” library, which resulted in quite low CVs on peptide and protein level (8 and 7% respectively), whereas the “Pulsar GS IS 15” analysis showed the highest peptide CV (10%) among the DIA analyses.

##### Specificity

Next, we evaluated the quantification data obtained by the different analyses regarding differential abundance detection specificity - *i.e.* the ability to differentiate between true positives (TPs, the spike-in proteins) and false positives (FPs, the mouse matrix). For this, a differential abundance test consisting of ANOVA with post hoc Tukey test was applied to the MS1 precursor peak areas for DDA and the MS2 extracted ion chromatogram peak areas for DIA data (details see Statistical Analysis). To maintain very high statistical significance, we filtered for a relatively strict corrected *p* value of 0.01. In all analyses spike-in proteins (TP) and C2C12 background proteins (FP) were found to be differentially abundant.

**Fig. 6.**
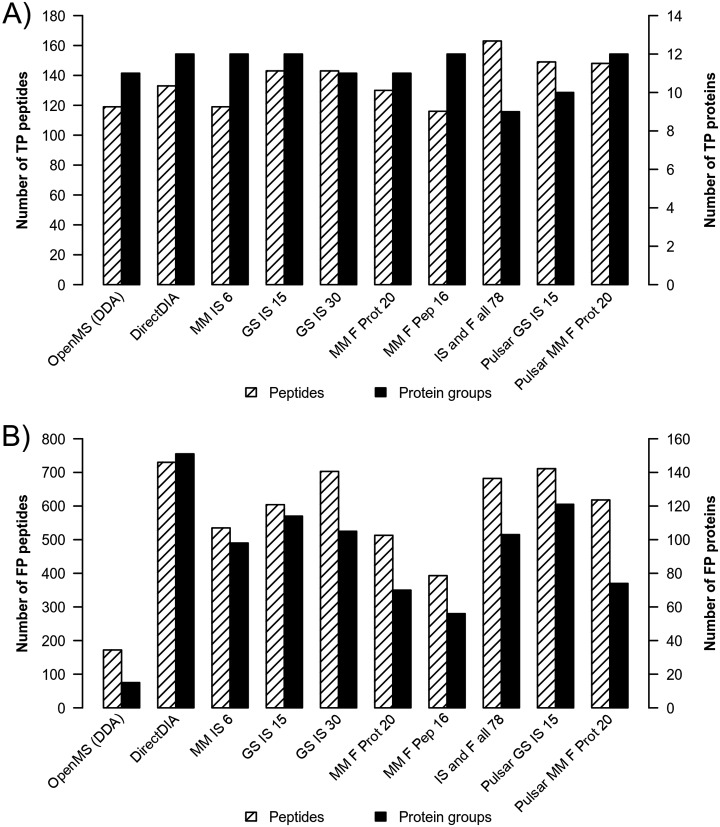
**Number of TP (*A*) and FP (*B*) proteins and peptides.** The numbers of proteins and peptides that passed the ANOVA *p* value filter of 0.01 (corrected for multiple testing) are shown for a) the 13 spike-in proteins (TPs) and b) mouse proteins (FPs). 9 (IS and F all 78) to 12 TP proteins and 116 (M Pep F 16) to 163 (IS and F all 78) TP peptides were detected as differentially abundant. The lowest number of FP on peptide and protein level was achieved with the DDA analysis whereas the highest number of differentially abundant mouse matrix proteins was detected using DirectDIA.

Of the 13 spike-in proteins, 9 to 12 were detected as differentially abundant in DDA or DIA analyses (see Fig. 6 and supplemental Table S10). The maximum of 12 TP detections was achieved by “DirectDIA,” “MM IS 6,” “GS IS 15,” “MM F Pep 16, and “Pulsar MM F Prot 20.” It must be considered, that though we quantified all proteins with each method (see Peptide and Protein Identification) not all passed the *p* value threshold. The differential abundance of the TP proteins was detected with all analyses, except for the one of the three lipases, myoglobin and alpha-synuclein (see also supplemental Table S11). In addition to the 13 TPs, we found spike-in contaminants to be differentially abundant (see supplemental Table S10). Because these were present in the samples, they were not counted as FPs. The lowest number of TP proteins was found with the “IS and F all 78” analysis that identified only 9 spike-in proteins as differentially abundant. At the same time the highest number of TP peptides (163) was detected, which cover all 13 spike-in proteins. The same was observed with the other DIA analyses. This indicates that the applied approach of taking the mean of the top three peptide intensities to infer protein quantities is insufficient.

Moreover, we analyzed the misdetection (FP) of C2C12 mouse proteins as differentially abundant. Surprisingly, with DDA the lowest number of FP (15 protein groups and 172 peptides) was achieved whereas DirectDIA resulted in the highest number of FP, both on protein (151) and peptide level (730, see Fig. 6 and supplemental Table S10). The library-based DIA analyses detected between 56 (“MM F Pep 16”) and 121 (“Pulsar GS IS 15”) FP protein groups, whereas the analyses using the fractionation libraries (“MM F Pep 16” and “MM F Prot 20”) exhibited the lowest number of FP, both on protein and peptide level.

To investigate why we found much more FP with DIA than with DDA we had a closer look at the quantification data. We found that most FP could be classified into two categories: group I showed differential abundance trends very similar to the spike-in proteins (see supplemental Fig. S3 for an example). The peptides of these “correlating” proteins most likely originated from one of the spike-in solutions but the corresponding non-mouse proteins were not included in our database and have shared peptides with (probably homolog) mouse proteins. Even though we performed the initial check for spike-in contaminants (see Statistical Analysis), we used an MS with lower resolution for this than the one used for the DIA experiment. Therefore, we might have missed some of the contaminants in the preliminary checks. The second and larger group (II) encompassed proteins exhibiting a very small fold-change over the five samples with usually only one sample exhibiting a slightly higher or lower abundance than the other four. These “low fold change” protein hits are probably found to be significantly differentially abundant because of a lower CV of DIA compared with DDA and statistical effects emerging from the limited number of replicates. In DDA these were not found to have a significant differential abundance at *p* < 0.01 (data not shown). Both groups were filtered out by A) a correlation analysis and B) requiring a minimal fold change of 1.3 (details see Statistical Analysis). After applying both filters to all analyses (DDA and DIA), the number of FP was reduced significantly in all DIA analyses, so that they now exhibited comparable values to DDA (see Table III). On peptide and protein level the lowest FP rates were achieved using the “GS IS 15” and “GS IS 30” libraries for DIA analyses, whereas the highest FP rates were observed with “Pulsar MM F Prot 20” and “MM F Pep 16.” The OpenSWATH analysis yielded in about the same numbers of TP and FP, both with the unfiltered and filtered approach. Therefore, a tool induced bias can be excluded.

**Table III:** False positive rate. Number of FP and resulting FP rate after correlation and low-fold change filtering on protein and peptide level

	Protein level		Peptide level	
	FP (filtered)	FP rate	FP (filtered)	FP rate
DDA	5	20.00%	124	42.61%
DirectDIA	13	28.89%	118	35.22%
MM IS 6	8	25.00%	119	40.61%
GS IS 15	5	14.29%	123	34.36%
GS IS 30	6	16.22%	125	32.81%
MM F Prot 20	14	29.79%	167	43.04%
MM F Pep 16	12	30.77%	129	43.58%
IS and F all 78	14	28.57%	226	42.56%
Pulsar GS IS 15	12	26.67%	188	40.26%
Pulsar MM F Prot 20	14	30.43%	214	43.50%

To further analyze the specificity of differential abundance detection, ROC (receiver operator characteristics) curves, displaying the number of TP against the number of FP of a q-value sorted list, together with the respective AUC (area under the curve) were calculated (see Table IV). The higher the AUC the better the specificity of the analysis, which means that the TP are detected with higher significance than the FP. We found specificities with AUCs ranging from 0.61 to 0.92 on peptide level and from 0.8. to 0.97 on protein level (where an AUC of 1.0 is the theoretically best specificity). The best specificities on protein level were achieved in DIA analyses using libraries with a high similarity to the actual samples: the “MM IS 6” (AUC: 0.97) and the “GS IS 15” (AUC: 0.95, see supplemental Fig. S4 for exemplary ROC curves) whereas “Pulsar MM F Prot 20” and “IS and F all 78” achieved almost equally good results (AUCs 0.94 and 0.93 respectively). DDA showed a medium specificity compared with all other analyses on protein level, whereas DIA with “MM F Pep 16” had the overall lowest specificity. “Pulsar MM F Prot 20” showed a very high specificity on protein level (AUC: 0.94), but not on peptide level (AUC: 0.63).

**Table IV:** AUCs and mean absolute percentage errors. Table showing the AUC of the ROCs and the mean absolute percentage error (MAPE) of the respective method on the peptide and protein level. The MAPE was calculated between the median of achieved and expected protein/peptide fold changes, on log2 transformed ratios. The values can be interpreted as deviation between the achieved and expected ratio in percent

	Peptide		Protein	
	AUC	MAPE	AUC	MAPE
DDA	0.61	65.20%	0.88	57.77%
DirectDIA	0.92	16.16%	0.87	21.67%
MM IS 6	0.85	18.45%	0.97	23.15%
GS IS 15	0.92	13.99%	0.95	22.84%
GS IS 30	0.88	17.08%	0.87	25.92%
MM F Prot 20	0.73	16.10%	0.82	21.41%
MM F Pep 16	0.68	15.39%	0.80	27.21%
IS and F all 78	0.63	15.12%	0.93	27.52%
Pulsar GS IS 15	0.76	13.78%	0.88	17.69%
Pulsar MM F Prot 20	0.63	12.91%	0.94	19.53%

##### Accuracy

**Fig. 7.**
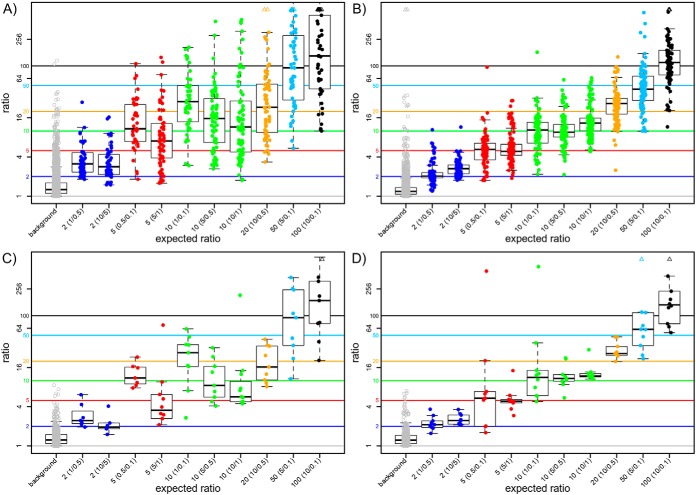
**Differential spike-in peptide and protein abundance ratios.** Detection of differential spike-in peptide (*A*, *B*) and protein (*C*, *D*) abundance ratios exemplarily DDA (*A*, *C*) and Pulsar GS IS 15 (*B*, *D*). Only the ratios passing the FC and correlation filter are plotted. The plots show the log2 transformed fold change ratios of peptides and protein groups for the spike-in proteins. The data is grouped by the states which are compared, *e.g.* for a fold change of 2 the spike-in states 1 pmol and 0.5 pmol and 10 pmol and 5 pmol. Plots for all other analyses can be found in the supplements (supplementary accuracy plots). It can be observed that the average deviation of the original values with DIA is smaller than with DDA.

Because the amount of spiked-in protein was known for every sample and each of the 13 spike-in proteins, the theoretical FCs can be compared with the relative quantification results of the DDA and DIA data analyses (see box plots in Fig. 7 and supplementary accuracy plots). The accuracy of differential abundance detection was assessed based on the mean absolute percentage error (MAPE, see 2.2.4). The MAPE varied greatly among the different analyses (see Table IV). DDA, although able to detect 74% of all possible differential states showed the highest deviation from the actual abundance ratios of spike-in proteins based on the MAPE (see Fig. 7*A* + 7*C*). The median FCs were more accurate with higher spike-in amounts, but higher deviations were observed with low spike-in amounts, especially for 0.1 pmol. For example, the fold-change of 10 was hit more accurate, when the spike-in amounts 10 pmol and 1 pmol were compared, whereas the ratios of 1 pmol to 0.1 pmol and 5 pmol to 0.5 pmol were less accurate. This trend was not observed in the DIA approach, indicating a higher accuracy and linear range - at least in the concentration range discussed here. The MAPE of all DIA analyses was much lower for all ratios and amounts, both on peptide and protein level, compared with DDA. The lowest deviation from the actual abundance ratios on peptide and protein level was observed with the Pulsar libraries (see Fig. 7*B* + 7*D* and Table IV) whereas “MM F Pep 16” and “IS and F all 78” exhibited the highest MAPE on protein level (supplementary accuracy plots). Besides the Pulsar libraries, DirectDIA and “MM F Prot 20” exhibited the best accuracies on protein level. In general, the accuracy on peptide level was better than on protein level.

In summary these quantification data show

- The larger the spectral library, the higher the CV on peptide and protein level—
- In DIA small FCs might entail the risk of more FP—
- Quantification performance of DIA is superior to DDA, especially in terms of reproducibility and accuracy—
- Quantification accuracy is decreased when considering low protein/peptide amounts in DDA, but not in DIA—
- Quantification on peptide level is preferable because of better differential abundance detection and higher accuracy—

##### Discussion

In this study, we compared the identification and especially the quantification characteristics of DDA and different DIA approaches including a spectral library-free DirectDIA analysis and various spectral library-based analyses. Among these, we used sample-specific libraries of differing complexity. As we performed the analysis with two independent software packages—Spectronaut and OpenSWATH—we could confirm, that the results were not specific for one tool. Even though both approaches yielded different numbers, the general trends were in the same directions. As our focus was the impact of different libraries, not the comparison of tools, we only give the numbers and values generated by the SN analysis in the following.

For a thorough comparison between the different analysis approaches we created the gold standard spike-in sample set (GS) mimicking characteristics of complex biological samples. The constant background of C2C12 cell lysate was spiked with physiological levels of 13 non-mouse proteins in varying ratios. The total spike-in amount was kept constant for all samples. The combination of the libraries and the GS sample set allowed a comprehensive in-depth evaluation of quantification performance of DIA in terms of reproducibility, specificity and accuracy.

Several studies already showed that spectral library-based DIA surpasses shotgun DDA in terms of number of identified peptides and proteins (1, 53) and has a significantly greater run-to-run identification overlap (54, 55). Here, we confirmed both points independent of the library complexity although especially peptide identification and consistency varied between the used spectral libraries. Using larger spectral libraries, either by combining many DDA runs (“GS IS 30”) or different sample preparation strategies (“IS and F all 78”) or by using a particular search engine (Pulsar), resulted in high numbers of extractions from DIA data (on average 4700). Smaller libraries of up to 15 repetitive DDA measurement of non-fractionated samples (“MM IS 6” or “GS IS 15” libraries) identified about 3800 protein groups. Sample fractionation for library creation on peptide or protein level, although enlarging library size, did not prove to be very beneficial in extracting DIA data. Fractionation on protein level (using the “MM F Prot 20” library) achieved more identifications than fractionation on peptide level (“MM F Pep 16”). Govaert *et al.* also observed that an increase in identifications from DIA data is not proportional to the increase in library size when sample prefractionation is used (29). The authors also showed that high-pH reverse phase peptide fractionation is less effective than SDS-PAGE protein fractionation in terms of number of identified peptides and proteins. Nonetheless, this technique is often applied in clinical studies to increase library depth (*e.g.* (56, 57)). When looking at the identification of specific proteins - here the spike-in proteins - a higher identification consistency over all 15 DIA runs was achieved with libraries generated from DDA data of non-fractionated samples (especially “Pulsar GS IS 15,” “GS IS 30,” and “GS IS 15”). In summary, we recommend using libraries from repetitive DDA measurements of individual samples or a sample pool for protein identification. The use of a sample pool is of advantage especially if only small sample amounts are available. As DirectDIA shows similar identification numbers as DDA, this might also be an option in case of very limited sample amounts as it is often the case in clinical studies. To maximize identifications, complex libraries deriving from different sample preparation strategies should be used.

In quantitative proteome studies a high quantification reproducibility is of utmost importance. In terms of peptide and protein quantification, DIA outperforms DDA reproducibility in this study. A similar superiority of DIA compared with DDA was already described in other studies (1, 16). Because of the stochastic nature of data-dependent Top10 acquisition and a higher interference when using MS1 peak areas for quantification, the reproducibility of DDA-based analyses is limited in general.

In addition, quantification specificity—the ability to clearly differentiate between regulated and unregulated proteins—greatly influences the outcome of differential proteome studies. Our GS data set is especially suited to assess the specificity of each analysis method based on the number of true (TP) and false positives (FP) as well as good discrimination of these as deduced from ROCs. We found, that although OpenMS analysis of DDA data led to an acceptable high number of TP peptides and proteins and low number of FP, the discrimination of both on peptide level was difficult. This means that when quantification data are sorted by *p* value the real candidates would not necessarily be the first on the list; making manual inspection and subsequent verification indispensable. Even when applying a relatively strict *p* value cutoff of 0.01, which was corrected for multiple testing, DIA exhibits many FP. The application of a FC filter (here we applied 1.3) drastically reduced the number of FP to 4–19% of the unfiltered value. Obviously, the applied correlation filter cannot be used in real life analyses, as the differentially expressed proteins and their ratios are not known.

Interestingly, in DIA data analysis we found that the used spectral library also had a great effect on the quantification specificity although raw data were the same. Altogether, using small libraries (“MM IS” and “GS IS”) resulted in the best discrimination between TP and FP both on peptide and protein level whereas the most complex libraries (“Pulsar MM F Prot 20” and “IS and F all 78”) led to lower specificity especially on peptide level. Using sample fractionation or Pulsar for library creation does not seem beneficial but also not very disadvantageous. It was also found by Wu *et al.* (28) that libraries should not be excessively large to achieve good quantification specificity.

Quantification accuracy, describing how well the detected FC matches the theoretical FC, was much higher in all DIA analyses compared with DDA. Especially when considering low protein, respectively peptide amounts the DDA accuracy was affected, independent from the FC itself. Only small differences in accuracy were observed between the various DIA analyses ranging from 13–18% on peptide and 18–28% on protein level (DDA: 65 and 58%, respectively). Here, the use of Pulsar seemed to be slightly beneficial resulting in the highest accuracies both with “Pulsar MM F Prot 20” and “Pulsar GS IS 15.” The most complex library “IS and F all 78” resulted in the lowest accuracy on protein level, whereas the smallest library “MM IS 6” exhibited the lowest accuracy on peptide level. These discrepancies between peptide and protein level quantifications point toward an insufficient protein inference. This becomes even more evident when inspecting the detection of TPs: whereas on protein level not all 13 spike-in proteins could be detected as differentially abundant, on peptide level all spike-in proteins could be successfully quantified. This highlights the importance of developing more accurate methods to infer protein quantities from peptide intensities.

Altogether, our analyses showed that DIA is superior to DDA. Using spectral libraries for DIA analysis always increases identification and quantification depth compared with DDA. When the aim of a study is only identification the use of large sample-specific libraries is beneficial, whereas for quantification smaller libraries perform better. The use of Pulsar seems to be a good compromise for both, identification and quantification. Nevertheless, its application in Spectronaut is debatable: the number of identifications and quantifications are raised substantially compared with other methods, but Pulsar has no publication of the underlying algorithms yet. Also, the FDR estimation in Pulsar is a black box in the used implementation, which makes deeper inspection and analysis of the correctness impossible. DirectDIA could be a good option when no libraries can be created, *e.g.* because of limited sample availability. In general, we recommend quantification and statistical analyses on peptide level for differential proteome studies.

## DATA AVAILABILITY

Data has been deposited to the ProteomeXchange repository (https://www.ebi.ac.uk/pride/archive) with identifiers PXD012986, PXD012987, PXD012988, and PXD014956.

Reviewer access data is provided in the respective document.

## Supplementary Material

Supplemental file 01

Supplemental file 02 - spike-in FASTA

Supplemental file 03 - additional plots

## Footnotes

^1^ The abbreviations used are:

DIA: data-independent acquisition
AAA: amino acid analysis
ACN: acetonitrile
AGC: automated gain control
AmBic: ammonium bicarbonate
ANOVA: analysis of variance
AUC: area under the curve
CV: coefficient of variation
DDA: data-dependent acquisition
DTT: dithiothreitol
FA: formic acid
FC: fold change
FP: false positive
F: fractionation
GS: gold standard
HCD: higher-energy collision dissociation
in-sol: in-solution
IS: in-solution
LC-MS/MS: liquid chromatography coupled tandem mass spectrometry
LFQ: label-free quantification
MAPE: mean absolute percentage error
MM: master mix
NCE: normalized collision energy
PD: proteome discoverer
Pep: peptide
Prot: protein
ROC: receiver operator characteristic
SN: spectronaut
SRM: selected reaction monitoring
TFA: trifluoroacetic acid
TP: true positive.
