# Supplementary material for: Reproducibility, Specificity and Accuracy of Relative Quantification Using Spectral Library-based Data-independent Acquisition
Source: Mol Cell Proteomics. 2019 Nov 7;19(1):181–97. doi: 10.1074/mcp.RA119.001714 (PMC6944235; doi:10.1074/mcp.RA119.001714)
Supplement: Supplemental file 01 [file 155027_1_supp_412482_pzgndj.pdf]

Supplement
Reproducibility, specificity and accuracy of relative quantification using spectral library-based data independent acquisition

Authors:

Sandra Pacharra[a+], Katalin Barkovits[a+], Kathy Pfeiffer[a], Simone Steinbach[a], Martin Eisenacher[a], Katrin Marcus*[a#] and Julian Uszkoreit*[a#]

Affiliations:

[a]Medizinisches Proteom-Center, Ruhr University Bochum, Faculty of Medicine, Bochum, Germany

*Corresponding authors: Julian Uszkoreit, Katrin Marcus

[+]these authors contributed equally to the work

[#]these authors contributed equally to the work

2 Materials and methods

Supplementary Table S1: Spike-in proteins and their physical and chemical properties: molecular weight (MW), isoelectric point (pI), and hydrophobicity (GRAVY score).

Protein	Species	UniProt Accession	MW (kDa)	pI	GRAVY score
α-synuclein	Homo sapiens	P37840	14.46	4.67	-0.403
β-lactoglobulin	Bos taurus	P02754	18.281	4.83	-0.162
Fibrinogen α	Homo sapiens	P02671	91.359	5.79	-0.885
Fibrinogen β	Homo sapiens	P02675	50.763	7.95	-0.849
Fibrinogen γ	Homo sapiens	P02679	48.483	5.24	-0.682
Glucose oxidase	Aspergillus niger	P13006	63.273	4.94	-0.241
Hemoglobin α	Homo sapiens	P69905	15.126	8.73	0.035
Hemoglobin β	Homo sapiens	P68871	15.867	6.81	0.001
Lipase 1	Candida rugosa	P20261	57.095	4.68	-0.056
Lipase 2	Candida rugosa	P32946	57.538	5.00	-0.068
Lipase 3	Candida rugosa	P32947	57.301	5.22	-0.047
Lysozyme C	Gallus gallus	P00698	14.313	9.32	-0.472
Myoglobin	Equus caballus	P68082	16.951	7.36	-0.396

Supplementary Table S2: Composition of samples with master mix samples (MM) for library generation.

	**MM IS 6**	**MM F Prot**	**MM F Pep**
Spike-in proteins (pmol)	3x1 and 3x5	12	6
C2C12 lysate (µg)	20	80	50

Supplementary Table S3: Overview of spectral library creation parameters regarding spike-in type (varying or constant spike-in amount), digest type (In-solution or in-gel digestion), fractionation level (protein or peptide), sample amount loaded per LC-MS/MS run vs. sample amount of total library, number of LC-MS/MS runs, time (effort of sample preparation combined with measurement time) and software (software tools used).

**Name**	**Spike-in type**	**Digest type**	**Fractionation**	**Sample per run (ng) / total (µg)**	**No of runs**	**Time (h)**	**Software**
MM IS 6	Constant	In-solution	No	200 ng<br>1.2 µg	6	39.5	PD+SN11
GS IS 15	Varying	In-solution	No	200 ng<br>3 µg	15	48.75	PD+SN11
GS IS 30	Varying	In-solution	No	200 ng<br>6 µg	30	97.5	PD+SN11
MM F Prot 20	Constant	In-gel	Protein	≤ 800 ng<br>11.28 µg	20	115	PD+SN11
MM F Pep 16	Constant	In-solution	Peptide	≤ 800 ng<br>9.94 µg	16	78	PD+SN11
IS and F all 78	Combined	Combined	Combined	≤ 800 ng<br>36.22 µg	78	349.5	PD+SN11
Pulsar GS IS 15	Varying	In-solution	No	200 ng<br>3 µg	15	48.75	SN Pulsar
Pulsar MM F Prot 20	Constant	In-gel	Protein	≤ 800 ng<br>11.28 µg	20	115	SN Pulsar

Supplementary Table S4: Parameters of DIA data analysis using SN 11 and SN Pulsar. Basically the default settings were used, except for the decoy fraction (0.5) and the enzyme (Trypsin). As the quantitative post analysis was performed externally, these settings are irrelevant.

Data Extraction	MS1 Mass Tolerance Strategy	Dynamic
	Correction Factor	1
	MS2 Mass Tolerance Strategy	Dynamic
	Correction Factor	1
XIC Extraction	XIC RT Extraction Window	Dynamic
	Correction factor	1
Calibration	Allow source specific iRT Calibration	True
	Calibration Mode	Automatic
	Precision iRT	True
	iRT <-> iRT Regression Type	Local (Non-Linear) Regression
	Calibration Carry-Over	False
Identification	Exclude Duplicate Assays	True
	Generate Decoys	True
	Decoy Method	Scrambled
	Decoy Limit Strategy	Dynamic
	Library Size Fraction	0.5
	Machine Learning	Per Run
	Precursor QValue Cutoff	0.01
	Protein QValue Cutoff	0.01
	Pvalue Estimator	Kernel Density Extimator
	Single Hit Definition	By Stripped Sequence
	Exclude Single Hit Proteins	False
Quantification	Interference Correction	True
	Min Fragment-Ions to Keep	3
	Min Precursor-Ions to Keep	2
	Proteotypicity Filter	None
	Major (Protein) Grouping	By Protein Group Id
	Minor (Peptide) Grouping	By Stripped Sequence
	Major Group Quantity	Mean peptide quantity
	Major Group Top N	True
	Max	3
	Min	1
	Minor Group Quantity	Mean precursor quantity
	Minor Group Top N	True
	Max	3
	Min	1
	Quantity MS-Level	MS2
	Quantity Type	Area
	Data Filtering	Qvalue
	Cross Run Normalization	True
	Normalization Strategy	Local Normalization
	Row Selection	Qvalue sparse
Workflow	Multi-Channel Workflow Definition	From Library Annotation

	Fallback Option	Labeled
	Profiling Strategy	None
	Unify Peptide Peaks	False
Protein Inference	Protein Inference Workflow	From protein-db matching
	Digest Type	Specific
	Enzymes/Cleavages Rules	Trypsin (others not selected)
	Toggle N-Terminal M	True
Post Analysis	Calculate Explained TIC	None
	Calculate Sample Correlation Matrix	False
	Differential Abundance Grouping	Major Group (Quantification Settings)
	Smallest Quantitative Unit	Precursor Ion (summed fragment ions)
	Differential Abundance Testing	Student's t-test
	Group-Wise Testing Correction	False
	Run Clustering	True
	Distance Metric	Manhattan Distance
	Linkage Strategy	Ward's Method
	Z-score transformation	False
	Gene Ontology	GO consortium go-basic
Reporting	(no automated reports created)	

3 Results

3.1 Spectral libraries

Supplementary Table S5: Number of precursors, peptides and protein groups included in the spectral libraries.

	Precursors	Peptides	Protein groups
**MM IS 6**	33,018	23,020	4,046
**GS IS 15**	34,329	24,457	4,126
**GS IS 30**	43,821	30,615	4,686
**MM F Prot 20**	58,983	41,322	5,859
**MM F Pep 16**	43,322	30,838	5,919
**IS and F all 78**	117,369	71,008	7,718
**Pulsar GS IS 15**	57,054	39,295	4,423
**Pulsar MM F Prot 20**	133,995	77,508	6,843

3.2 Peptide and protein identification

Supplementary Table S6: This table shows the number of peptides and protein groups in the respective spectral libraries (“library”), how many of these were extracted from GS data using a false discovery rate of 1% (“ident”) and how many of the identified peptides/protein groups were uniquely found with only the used method/library. The table gives an overview of the following graphs.

	**peptides**			**protein groups**		
	library	ident	unique	library	ident	unique
**DDA**	-	18,632	428	-	3,073	10
**DirectDIA**	-	21,394	438	-	2,979	50
**MM IS 6**	23,020	22,397	217	4,046	3,757	44
**GS IS 15**	24,457	23,911	110	4,126	3,938	47
**GS IS 30**	30,615	30,086	389	4,686	4,522	110
**MM F Prot 20**	41,322	27,724	587	5,859	4,081	43
**MM F Pep 16**	30,838	21,547	751	5,919	3,729	45
**IS and F all 78**	71,008	47,646	3,431	7,718	5,044	205
**Pulsar GS IS 15**	39,295	37,873	2,652	4,423	4,339	97
**Pulsar MM F Prot 20**	77,508	45,276	3,976	6,843	4,703	162

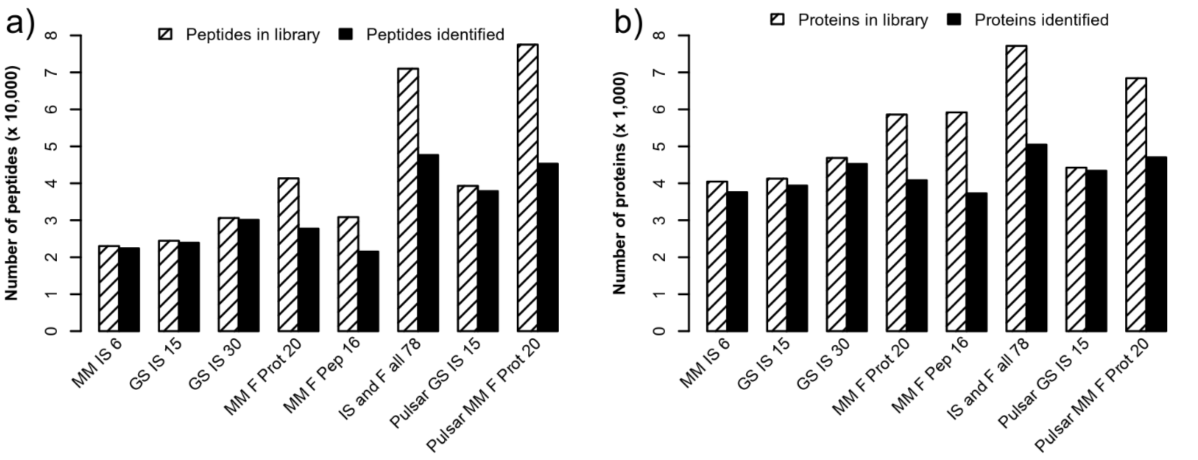

Figure S1a+b: Library recovery. DIA identification results compared to spectral library content regarding number of peptides (a) and number of proteins (b). While the simple IS libraries have a high library recovery rate, the libraries containing fractionated sample MS analyses exhibit 58-70% recovery.

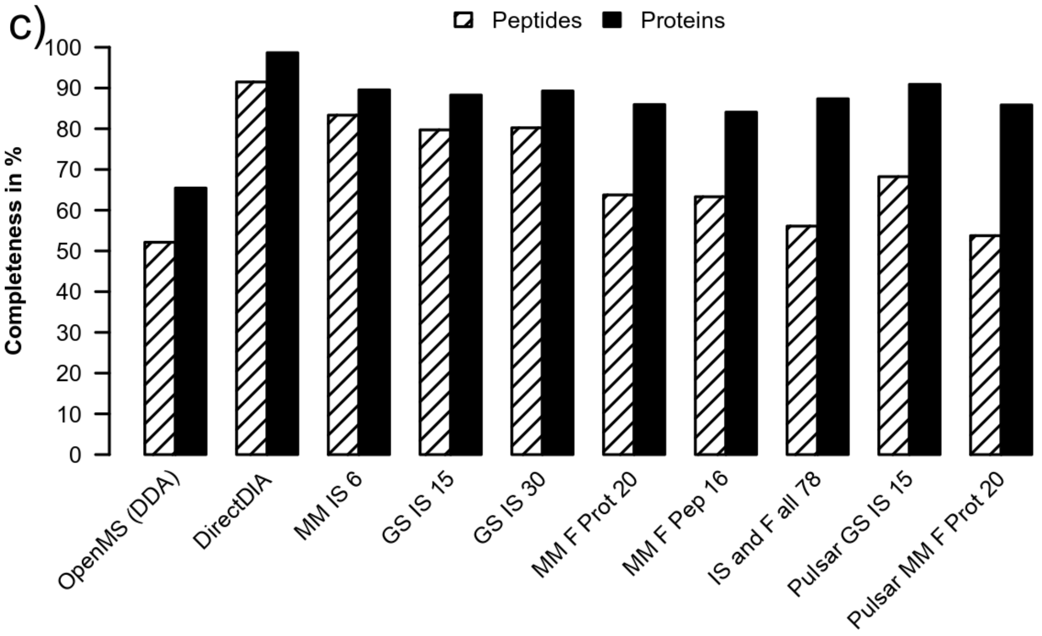

Figure S1c: Data completeness. Comparison of the portion of consistently identified peptides and proteins.

Supplementary Table S7: Recall rate of the spike-in proteins on identification basis. This table shows in how many of the 15 runs the respective protein was identified and quantifiable. Only the proteins, which were not identified in each run and method, are shown here.

	**Lipase 1 P20261**	**Lipase 2 P32946**	**Lipase 3 P32947**	**α-synuclein P37840**	**Myoglobin P68082**
**DDA**	13	9	7	14	15
**DirectDIA**	15	15	15	15	15
**MM IS 6**	15	11	12	12	15
**GS IS 15**	15	10	14	12	15
**GS IS 30**	15	11	15	13	15
**MM F Prot 20**	14	8	13	12	15
**MM F Pep 16**	14	7	9	12	15
**IS and F all 78**	14	10	13	12	14
**Pulsar GS IS 15**	14	14	14	13	15
**Pulsar MM F Prot 20**	12	9	13	12	15

a)

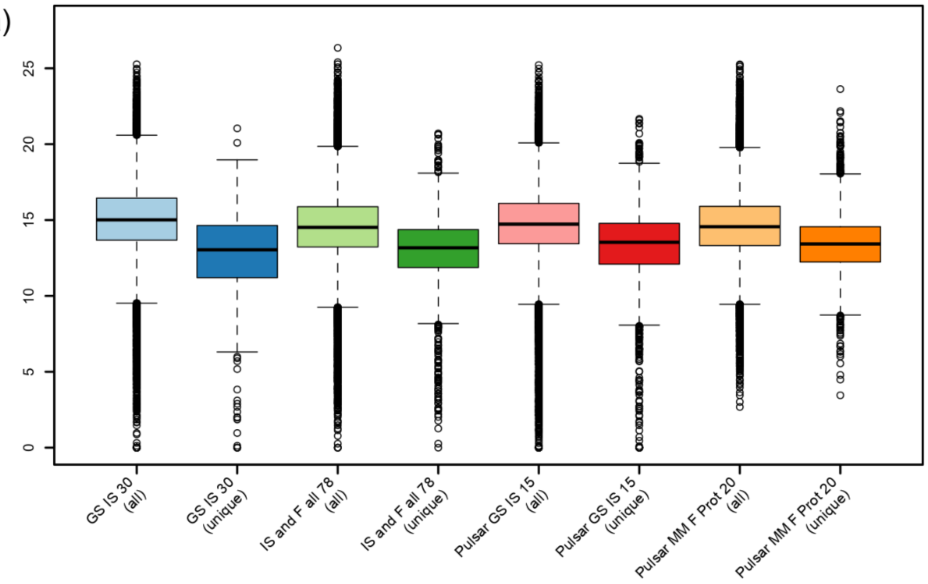

b)

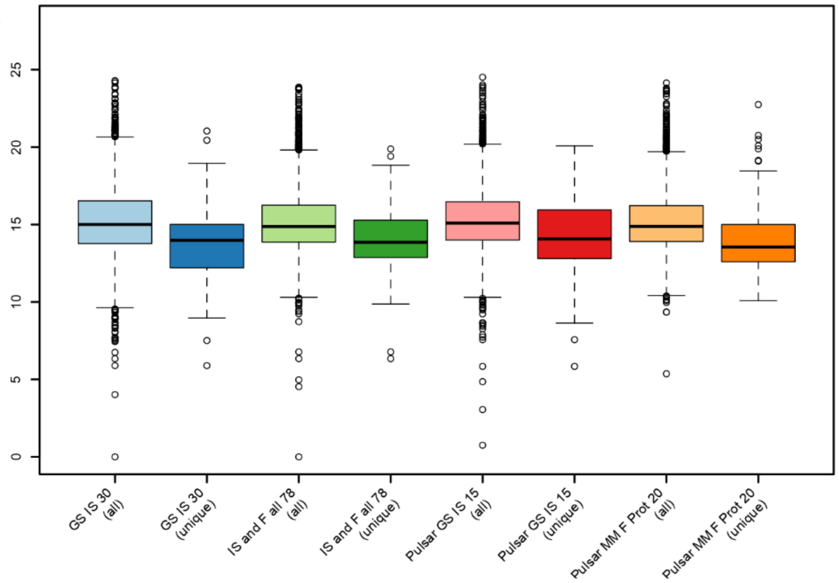

Supplementary Figure S2: Log2 transformed abundance values of selected analyses on (a) peptide and (b) protein group level. In the plots the values of all peptides/ protein groups, which were identified using the specified spectral library, are compared to the abundance of peptides/protein groups uniquely identified with the respective spectral library(after overlap analysis). All analyses show a clear shift to lower abundance values of unique identifications.

Supplementary Table S8: Percent completeness of Identifications. This table shows the percentage of protein groups and peptides, which were found in all and at least 80%, 66% and 50% of the runs.

	Peptides				Proteins			
	**100%**	**80%**	**66%**	**50%**	**100%**	**80%**	**66%**	**50%**
**DDA**	52	66	71	76	65	76	80	84
**DirectDIA**	91	93	94	94	99	99	99	99
**MM IS 6**	83	91	92	94	90	96	97	98
**GS IS 15**	80	86	87	88	88	93	94	95
**GS IS 30**	80	87	89	90	89	95	96	97
**MM F Prot 20**	64	74	77	80	86	94	96	97
**MM F Pep 16**	63	73	77	80	84	92	95	96
**IS and F all 78**	56	68	72	76	87	95	97	97
**Pulsar GS IS 15**	68	78	81	84	91	95	96	97
**Pulsar MM F Prot 20**	54	66	70	74	86	94	96	98

3.3 Peptide and protein quantification

Supplementary Table S9: Median peptide and protein group CVs of the various analyses in percent.

	**Peptide CV**	**Protein CV**
**DDA**	29.4%	24.3%
**DirectDIA**	6.3%	4.6%
**MM IS 6**	8.1%	6.6%
**GS IS 15**	7.6%	6.7%
**GS IS 30**	8.2%	6.9%
**MM F Prot 20**	7.7%	7.7%
**MM F Pep 16**	7.9%	9.0%
**IS and F all 78**	9.5%	8.4%
**Pulsar GS IS 15**	10.0%	7.0%
**Pulsar MM F Prot 20**	9.1%	9.1%

Supplementary Table S10: Overview on differential abundance detection on protein and peptide level at a p-value filter of 0.01. TP are the 13 spike-in proteins, spike-in contaminants are other proteins present in the spike-in solutions (as described in the methods section). FP are mouse matrix proteins detected as differentially abundant although they were actually present in equal amounts in all samples. Filtered means remaining FP after applying correlation analysis and the low FC filter of 1.3.

	Proteins				Peptides			
	TP	spike-in contaminants	FP	FP (filtered)	TP	spike-in contaminants	FP	FP (filtered)
**DDA**	11	9	15	5	119	48	172	124
**DirectDIA**	12	20	151	13	133	84	730	118
**MM IS 6**	12	12	98	8	119	55	535	119
**GS IS 15**	12	18	114	5	143	92	604	123
**GS IS 30**	11	20	105	6	143	113	703	125
**MM F Prot 20**	11	22	70	14	130	91	513	167
**MM F Pep 16**	12	15	56	12	116	51	393	129
**IS and F all 78**	9	26	103	14	163	142	682	226
**Pulsar GS IS 15**	10	23	121	12	149	130	711	188
**Pulsar MM F Prot 20**	12	20	74	14	148	130	618	214

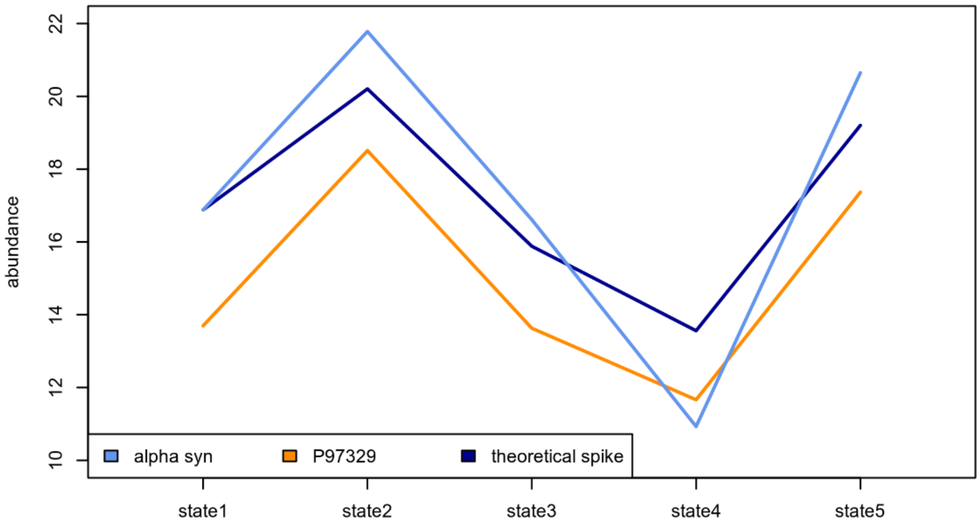

Supplementary Figure S3: Highly correlated abundance of protein to spike-in. The figure depicts the abundance profile of α-synuclein as it was measured in the “GS IS 30” analysis and the theoretical values. Additionally, the profile for protein P97329 (murine Kinesin-like protein KIF20A) is shown, which has a highly correlated abundance profile and was filtered out in the analysis.

Supplementary Table S11: Detection of spike-in differential abundance. The table shows, which spike-in proteins were found to be differentially abundant with the given methods. Only the proteins that were not found in all approaches are shown.

	Lipase 1	Lipase 2	Lipase 3	α-synuclein	Myoglobin
**DDA**		+	+		+
**DirectDIA**	+	+	+		+
**MM IS 6**	+		+	+	+
**GS IS 15**	+	+		+	+
**GS IS 30**			+	+	+
**MM F Prot 20**		+		+	+
**MM F Pep 16**		+	+	+	+
**IS and F all 78**				+	
**Pulsar GS IS 15**				+	+
**Pulsar MM F Prot 20**	+	+		+	+

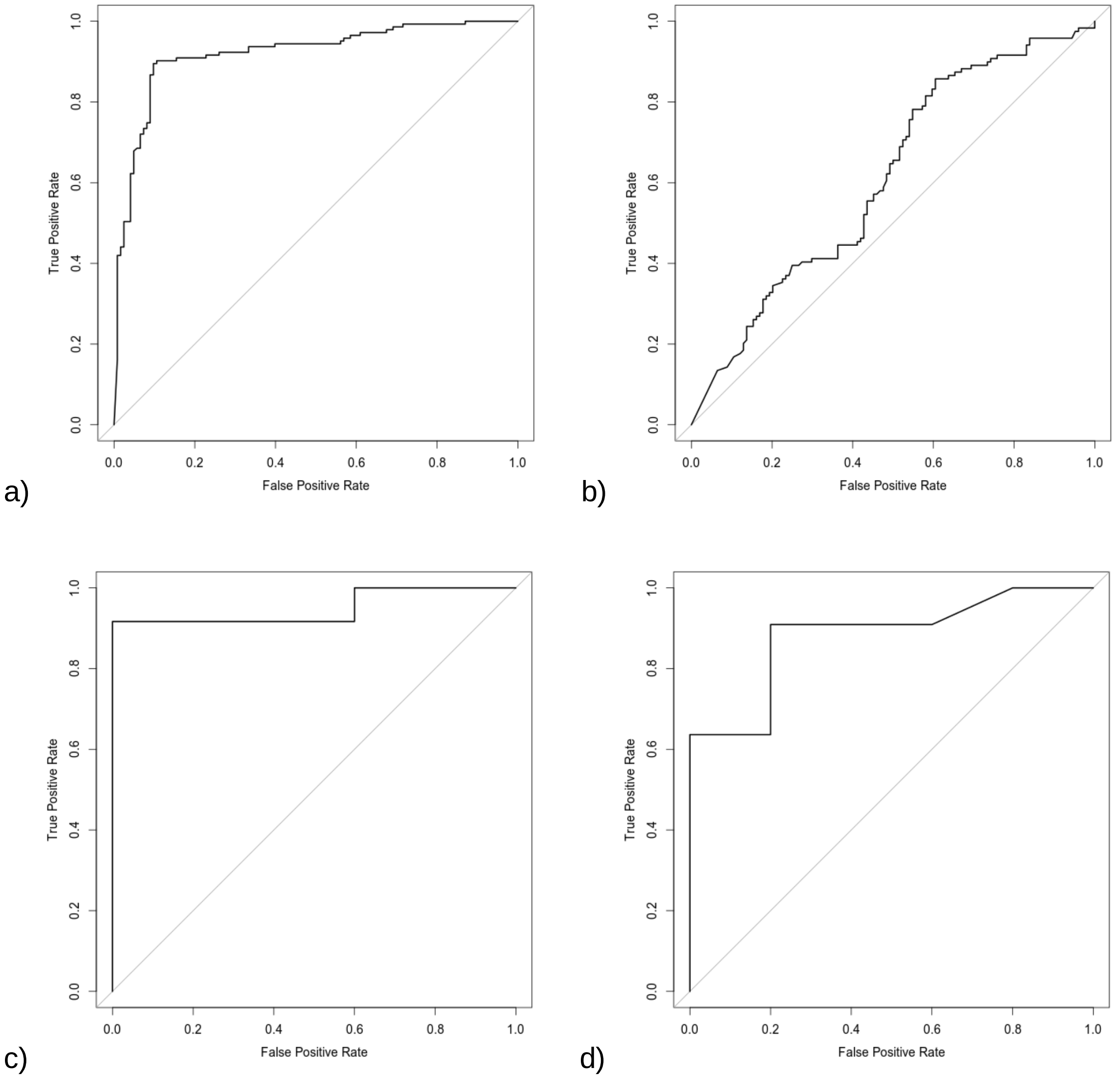

Supplementary Figure S3: ROC curves of the peptide (a+b) and protein (c+d) quantifications (sorted by increasing p-value) after correlation and low FC filtering. The plots show the data of “GS IS 15” (a+c) and DDA (b+d) analyses, which had the highest respectively lowest area under the curve (AUC) of all analyzed methods on the peptide level.

4 OpenSWATH Analysis of the Dataset

4.1 Generation of spectral libraries:

The following commands with the respective parameters were performed on the Mascot search results. For the actual Mascot identification, the same parameters as for the Spectronaut / ProteomeDiscoverer approach were applied.

4.1.1 PeptideProphet

```
docker run --rm -v /path/to/files/:/data/ biocontainers/tpp:v5.2_cv1 \
      xinteract -THREADS=8 -eT -ds -OAPd \
      -Ninteract.mascot.pep.xml \
      -DRP_mus_musculus_with_spikes_and_iRT_decoy_20180119.fasta \
      QExHF*.dat
```

4.1.2 iProphet

```
docker run --rm -v /path/to/files/:/data/ biocontainers/tpp:v5.2_cv1 \
      InterProphetParser DECOY=s interact.mascot.pep.xml iProphet.pep.xml
```

4.1.3 Mayu (FDR estimation)

```
docker run --rm -v /path/to/files/:/data/ biocontainers/tpp:v5.2_cv1 \
      perl /usr/local/tpp/bin/Mayu.pl -A iProphet.MM_IS_6.pep.xml \
      -C RP_mus_musculus_with_spikes_and_iRT_decoy_20180119.fasta \
      -E s -G 0.01 -H 101 -I 2
```

After Mayu, the tutorial states to get the score at protFDR just below 0.01 (in the csv file) for further filtering

4.1.4 SpectraST:

```
docker run --rm -v /path/to/files/:/data/ biocontainers/tpp:v5.2_cv1 \
      spectrast -cNSpecLib -cIHCD -cf'Protein!~s | Protein=~Biognosys' \
      -cP0.680737 \
      -c_IRT/data/irtkit_tutorial.txt -c_IRR iProphet.pep.xml

docker run --rm - v /path/to/files/:/data/ biocontainers/tpp:v5.2_cv1 \
      spectrast -cNSpecLib_cons -cIHCD -cAC SpecLib.splib
```

```
spectrast2tsv.py -l 300,1800 -s b,y -x 1,2 -o 3 -n 6 -p 0.05 -d -e \
      -w swath_windows_noheader.txt -k openswath \
      -a SpecLib_cons_openswath.tsv SpecLib_cons.sptxt
```

Then further in KNIME using the OpenSWATH (OpenMS nodes): convert library to TraML and create decoys

4.2 OpenSWATH identification and quantification

4.2.1 OpenSWATH

OpenSWATH analysis (OpenSWATH workflow) was applied on each DIA file separately using the TraML spectral library and a TraML file for the iRT peptides found in the respective library.

4.2.2 PyProphet (legacy)

run on the OpenSWATH output TSV files:

```
for file in *.tsv; do \
      pyprophet --d_score.cutoff="1" --ignore.invalid_score_columns $file;\
done
```

4.2.3 TRIC:

```
feature_alignment.py \
      --in  QExHF03751_with_dscore_filtered.csv \
      QExHF03753_with_dscore_filtered.csv \
      QExHF03755_with_dscore_filtered.csv \
[…]
      QExHF03779_with_dscore_filtered.csv \
      --out feature_alignment_pyProphet_TRIC_lowess_localMST.tsv \
      --file_format openswath --method LocalMST --max_rt_diff 30 \
      --target_fdr 0.01 --max_fdr_quality 0.05 \
      --mst:useRTCorrection True --mst:Stdev_multiplier 3.0 \
```

```
	--alignment_score 0.0001 --realign_method lowess \
	--matrix_output_method full \
	--dscore_cutoff 1.0 --frac_selected 0 --disable_isotopic_grouping \
	--out_matrix
feature_alignment_pyProphet_TRIC_lowess_localMST_outmatrix.tsv \
	--out_meta feature_alignment_pyProphet_TRIC_lowess_localMST_meta.tsv
```

4.3 OpenSWATH Results

Supplementary Table S5osw: Number of precursors, peptides and protein groups included in the spectral libraries.

	**OpenSWATH**		
	Precursors	Peptides	Protein groups
**MM IS 6**	26,253	20,520	3,573
**GS IS 15**	16,579	14,013	2,615
**GS IS 30**	30,666	23,743	3,664
**MM F Prot 20**	74,644	51,681	5,594
**MM F Pep 16**	81,069	56,380	6,677
**IS and F all 78**	112,759	71,892	6,853
**Pulsar GS IS 15**	-	-	-
**Pulsar MM F Prot 20**	-	-	-

Supplementary Table S6osw: This table shows the number of peptides and protein groups in the respective spectral libraries (“library”), how many of these were extracted from GS data using a false discovery rate of 1% (“ident”) and how many of the identified peptides/protein groups were uniquely found with only the used method/library. The table gives an overview of the following graphs.

**OpenSWATH**	**peptides**			**protein groups**		
	library	ident	unique	library	ident	unique
**MM IS 6**	20,520	17,438	372	3,573	3,158	14
**GS IS 15**	14,013	13,355	265	2,615	2,562	8
**GS IS 30**	23,743	21,220	478	3,664	3,408	20
**MM F Prot 20**	51,681	26,654	902	5,594	4,160	63
**MM F Pep 16**	56,380	28,673	907	6,677	4,539	93
**IS and F all 78**	71,892	32,568	1,245	6,853	4,681	46

Supplementary Table S7osw: Recall rate of the spike-in proteins on identification basis. This table shows in how many of the 15 runs the respective protein was identified and quantifiable. Only the proteins, which were not identified in each run and method, are shown here.

OpenSWATH	Lipase 1 P20261	Lipase 2 P32946	Lipase 3 P32947	α-synuclein P37840	Myoglobin P68082
**MM IS 6**	12	12	13	12	13
**GS IS 15**	12	7	15	12	14
**GS IS 30**	12	11	14	12	13
**MM F Prot 20**	12	13	13	12	13
**MM F Pep 16**	12	13	13	12	15
**IS and F all 78**	12	11	12	12	13

Supplementary Table S8osw: Percent completeness of Identifications. This table shows the percentage of protein groups and peptides, which were found in all and at least 80%, 66% and 50% of the runs.

OpenSWATH	Peptides				Proteins			
	**100%**	**80%**	**66%**	**50%**	**100%**	**80%**	**66%**	**50%**
**MM IS 6**	67	81	85	89	80	88	91	93
**GS IS 15**	75	87	90	93	87	93	95	97
**GS IS 30**	66	79	83	86	82	89	92	94
**MM F Prot 20**	58	73	78	83	77	86	89	92
**MM F Pep 16**	50	71	77	82	73	84	88	90
**IS and F all 78**	53	69	75	80	76	85	88	91

Supplementary Table S9osw: Median peptide and protein group CVs of the various analyses in percent.

	**OpenSWATH**	
	**Peptide CV**	**Protein CV**
**DDA**	-	-
**DirectDIA**	-	-
**MM IS 6**	9.3%	8.9%
**GS IS 15**	8.4%	7.5%
**GS IS 30**	9.0%	8.5%
**MM F Prot 20**	9.7%	9.5%
**MM F Pep 16**	10.2%	10.7%
**IS and F all 78**	10.2%	10.3%
**Pulsar GS IS 15**	-	-
**Pulsar MM F Prot 20**	-	-

Supplementary Table S10osw: Overview on differential abundance detection on protein and peptide level at a p-value filter of 0.01. TP are the 13 spike-in proteins, spike-in contaminants are other proteins present in the spike-in solutions (as described in the methods section). FP are mouse matrix proteins detected as differentially abundant although they were actually present in equal amounts in all samples. Filtered means remaining FP after applying correlation analysis and the low FC filter of 1.3.

OpenSWATH	Proteins				Peptides			
	TP	spike-in contaminants	FP	FP (filtered)	TP	spike-in contaminants	FP	FP (filtered)
**MM IS 6**	11	11	93	12	94	44	533	230
**GS IS 15**	12	7	86	11	102	40	480	192
**GS IS 30**	10	14	115	22	119	81	666	292
**MM F Prot 20**	10	9	81	14	117	74	653	331
**MM F Pep 16**	11	16	83	13	89	63	485	235
**IS and F all 78**	11	17	90	21	130	89	720	399

Supplementary Table S11osw: Detection of spike-in differential abundance. The table shows, which spike-in proteins were found to be differentially abundant with the given methods. Only the proteins that were not found in all approaches are shown.

**OpenSWATH**	Lipase 1	Lipase 2	Lipase 3	α-synuclein	Myoglobin
**MM IS 6**	+	+		+	
**GS IS 15**	+	+	+	+	
**GS IS 30**	+			+	
**MM F Prot 20**	+			+	
**MM F Pep 16**	+			+	+
**IS and F all 78**	+		+	+	
