# Supplementary figures and images for: Reproducibility, Specificity and Accuracy of Relative Quantification Using Spectral Library-based Data-independent Acquisition
Source: Mol Cell Proteomics. 2019 Nov 7;19(1):181–97. doi: 10.1074/mcp.RA119.001714 (PMC6944235; doi:10.1074/mcp.RA119.001714)

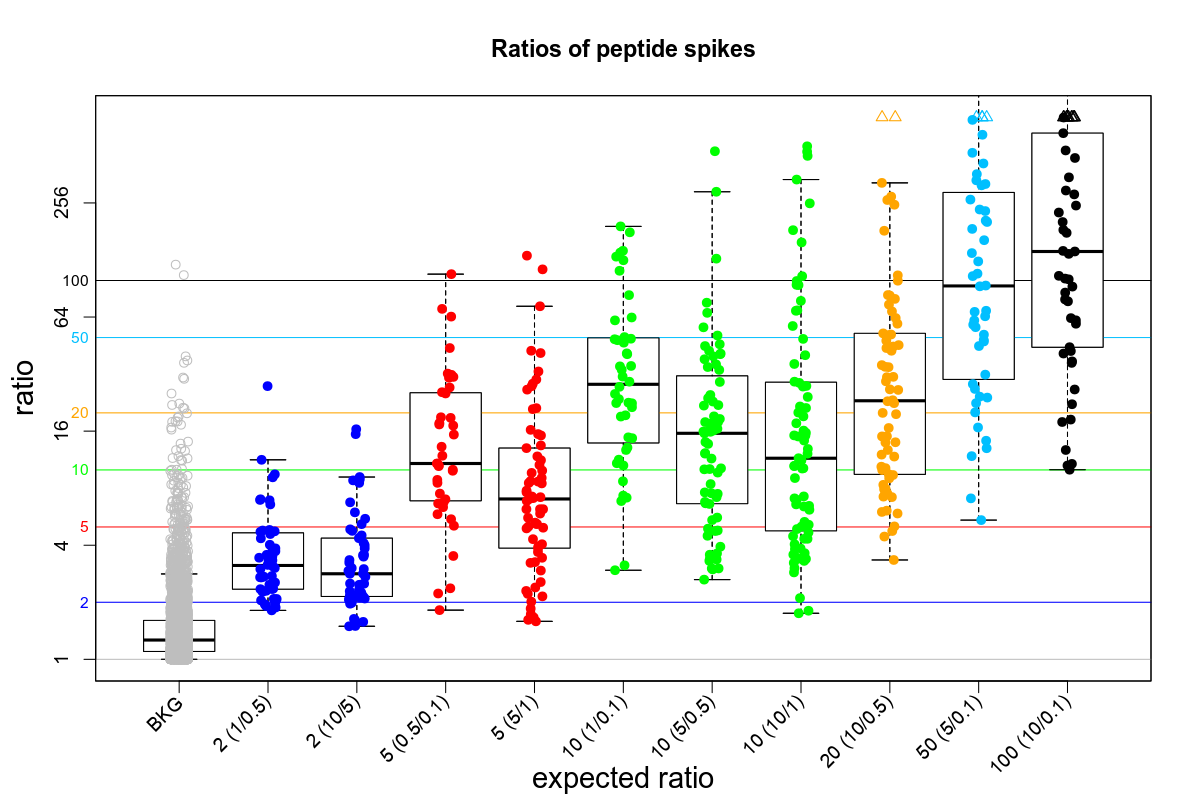

Supplement: Supplemental file 03 - additional plots [file 155027_1_supp_412484_pzgndk.zip › accuracy_plots/01-DDA-peptides.png]

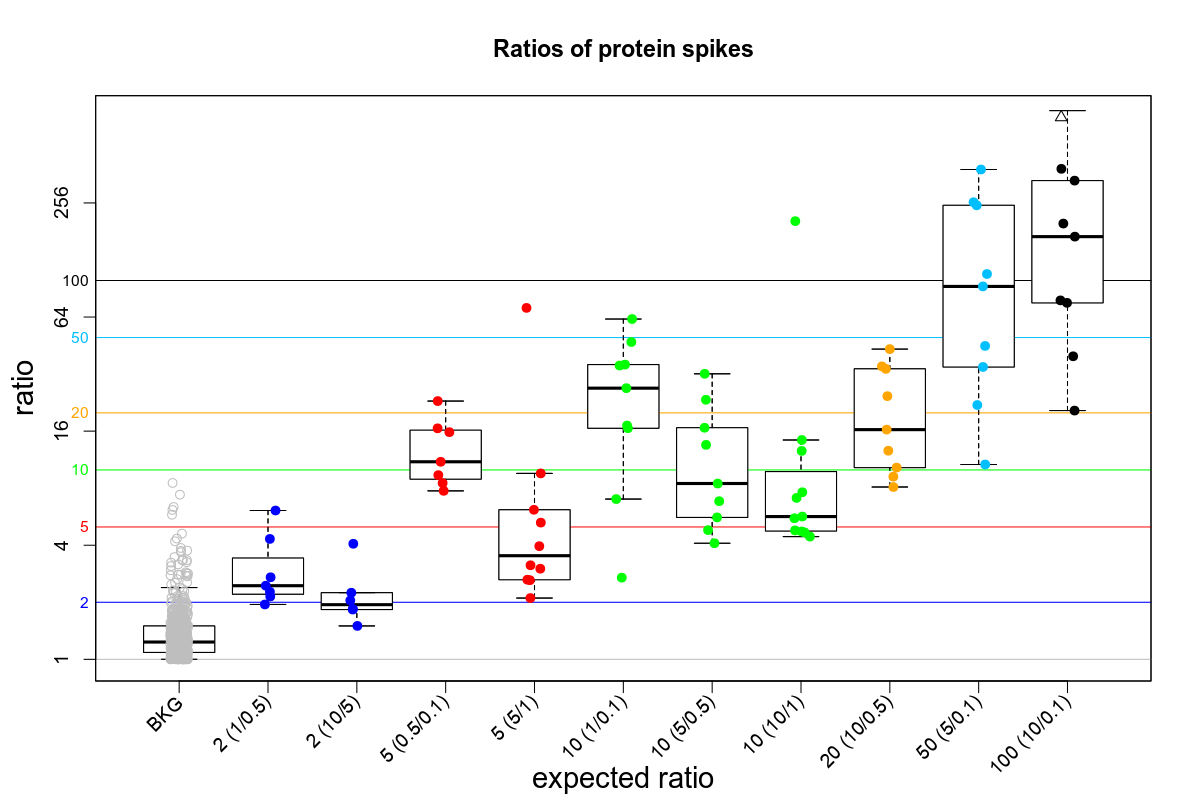

Supplement: Supplemental file 03 - additional plots [file 155027_1_supp_412484_pzgndk.zip › accuracy_plots/01-DDA-proteins.png]

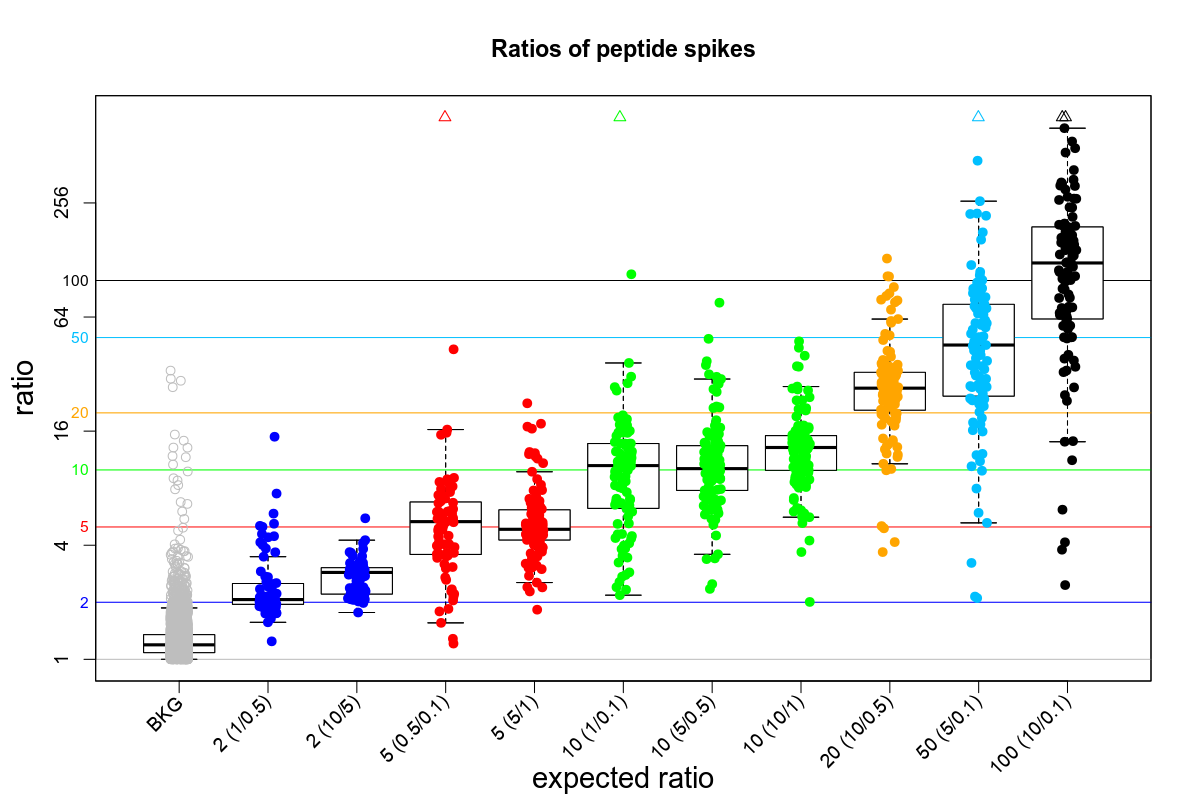

Supplement: Supplemental file 03 - additional plots [file 155027_1_supp_412484_pzgndk.zip › accuracy_plots/02-DirectDIA-peptides.png]

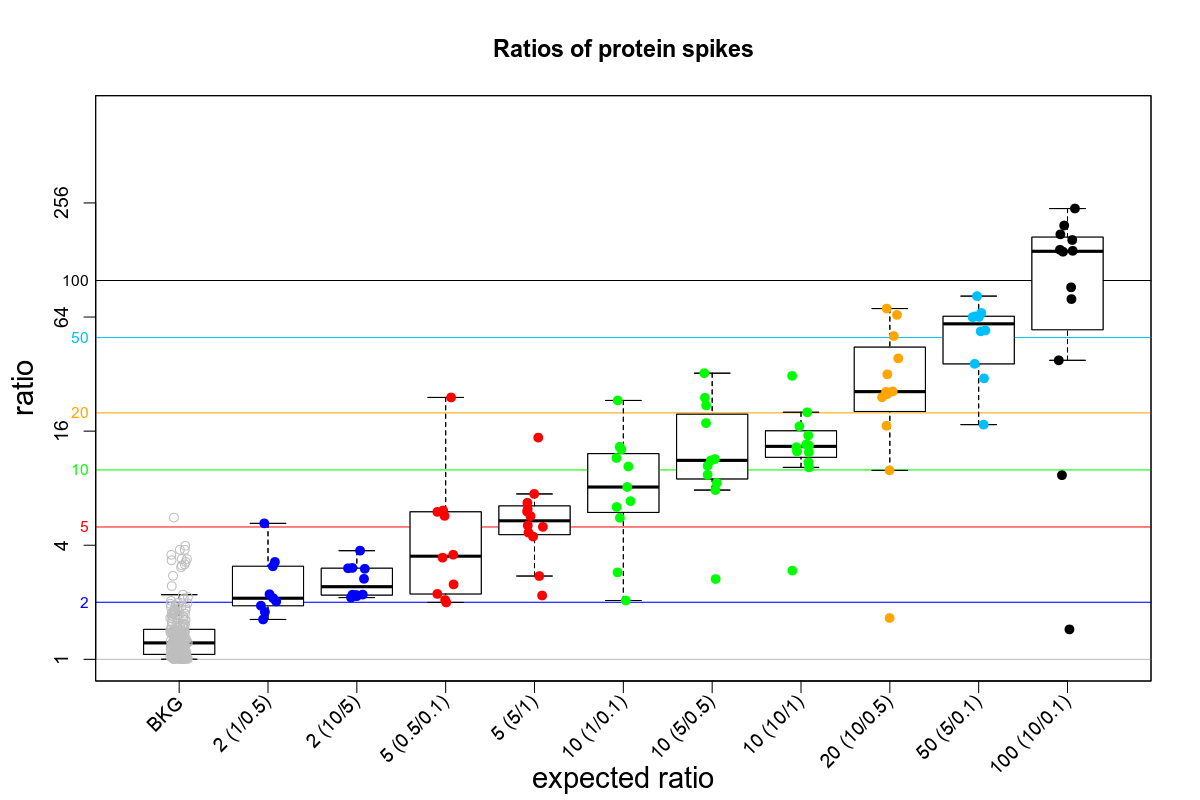

Supplement: Supplemental file 03 - additional plots [file 155027_1_supp_412484_pzgndk.zip › accuracy_plots/02-DirectDIA-proteins.png]

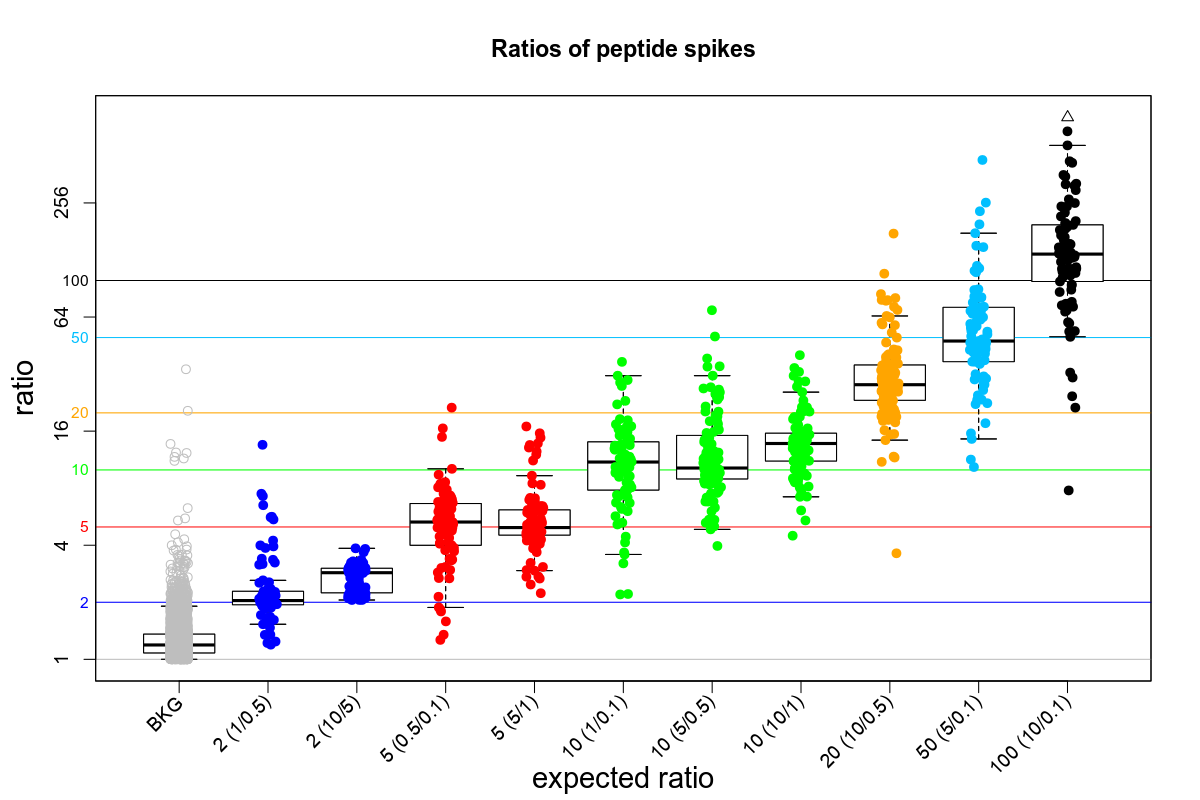

Supplement: Supplemental file 03 - additional plots [file 155027_1_supp_412484_pzgndk.zip › accuracy_plots/03-MM_IS_6-peptides.png]

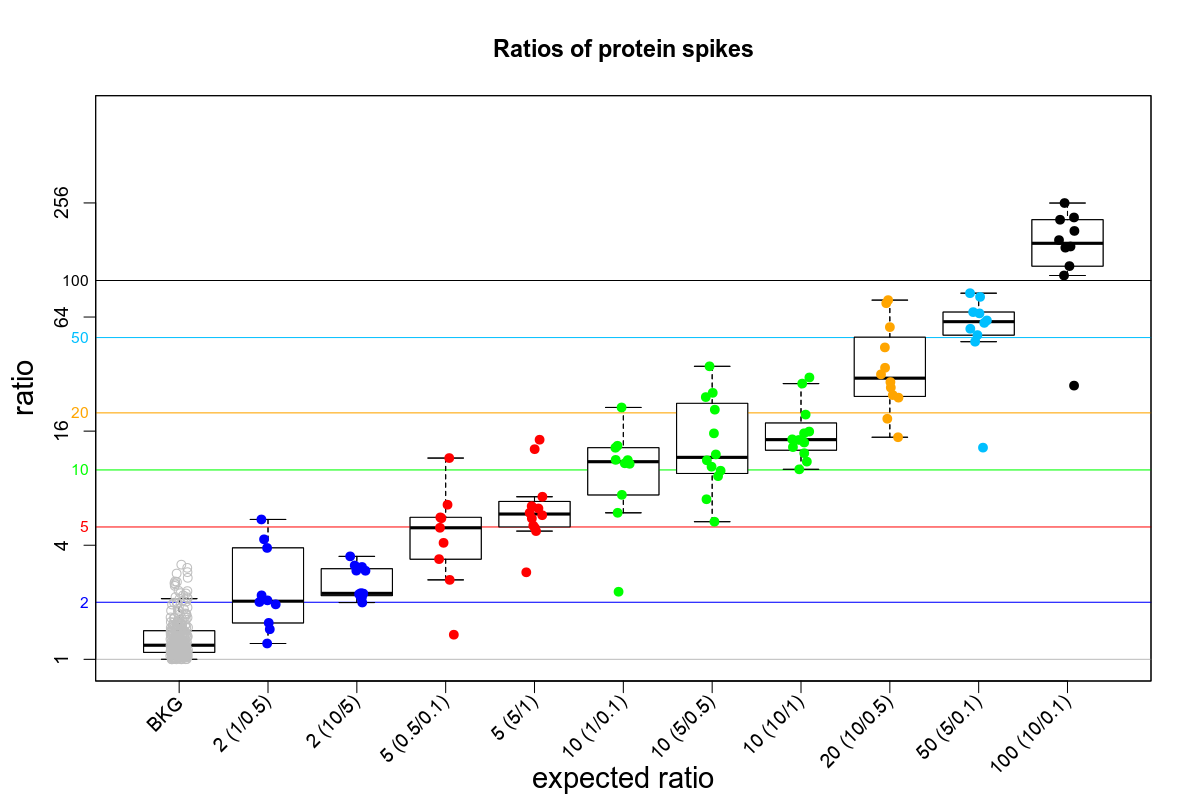

Supplement: Supplemental file 03 - additional plots [file 155027_1_supp_412484_pzgndk.zip › accuracy_plots/03-MM_IS_6-proteins.png]

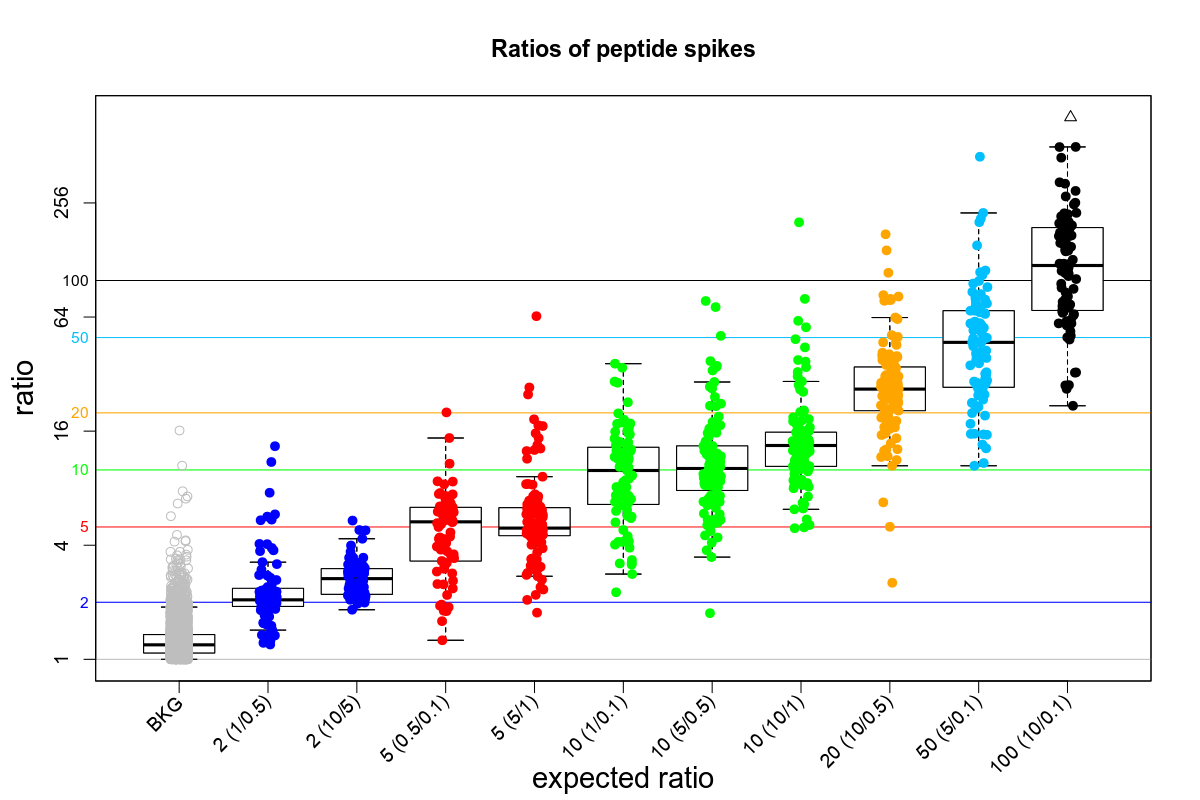

Supplement: Supplemental file 03 - additional plots [file 155027_1_supp_412484_pzgndk.zip › accuracy_plots/04-GS_IS_15-peptides.png]

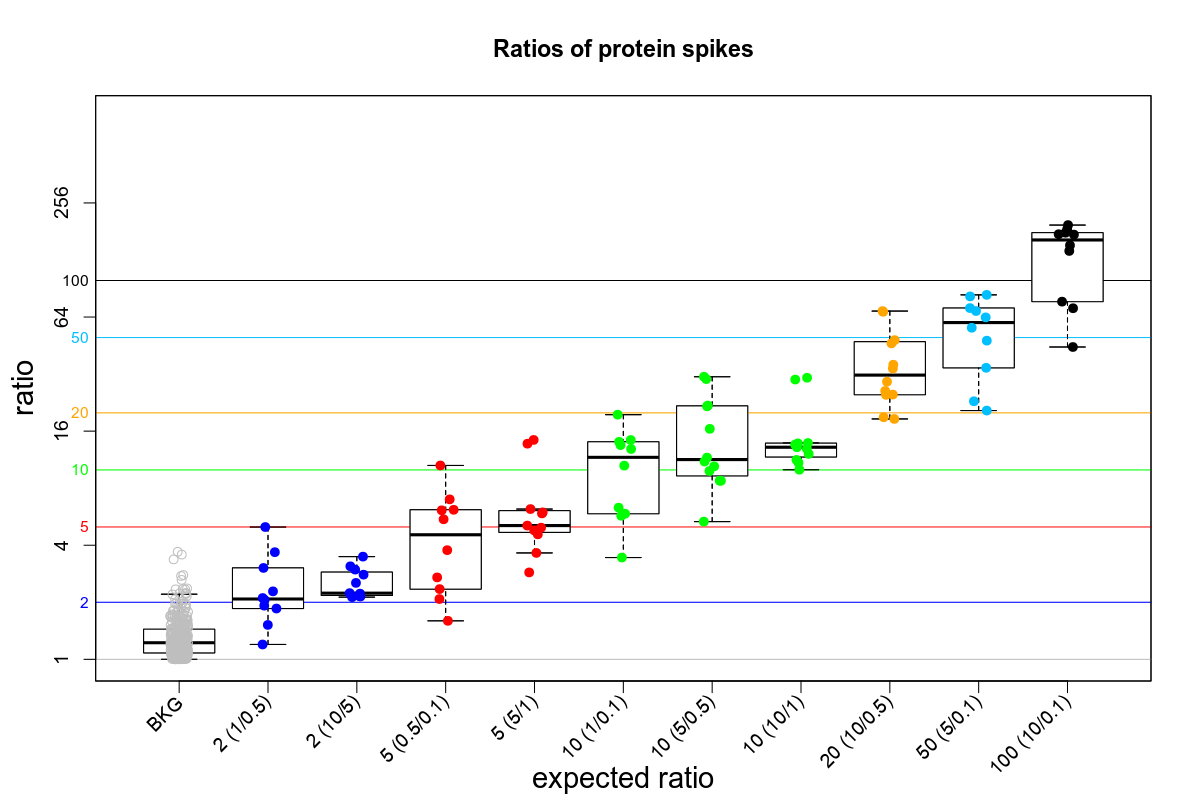

Supplement: Supplemental file 03 - additional plots [file 155027_1_supp_412484_pzgndk.zip › accuracy_plots/04-GS_IS_15-proteins.png]

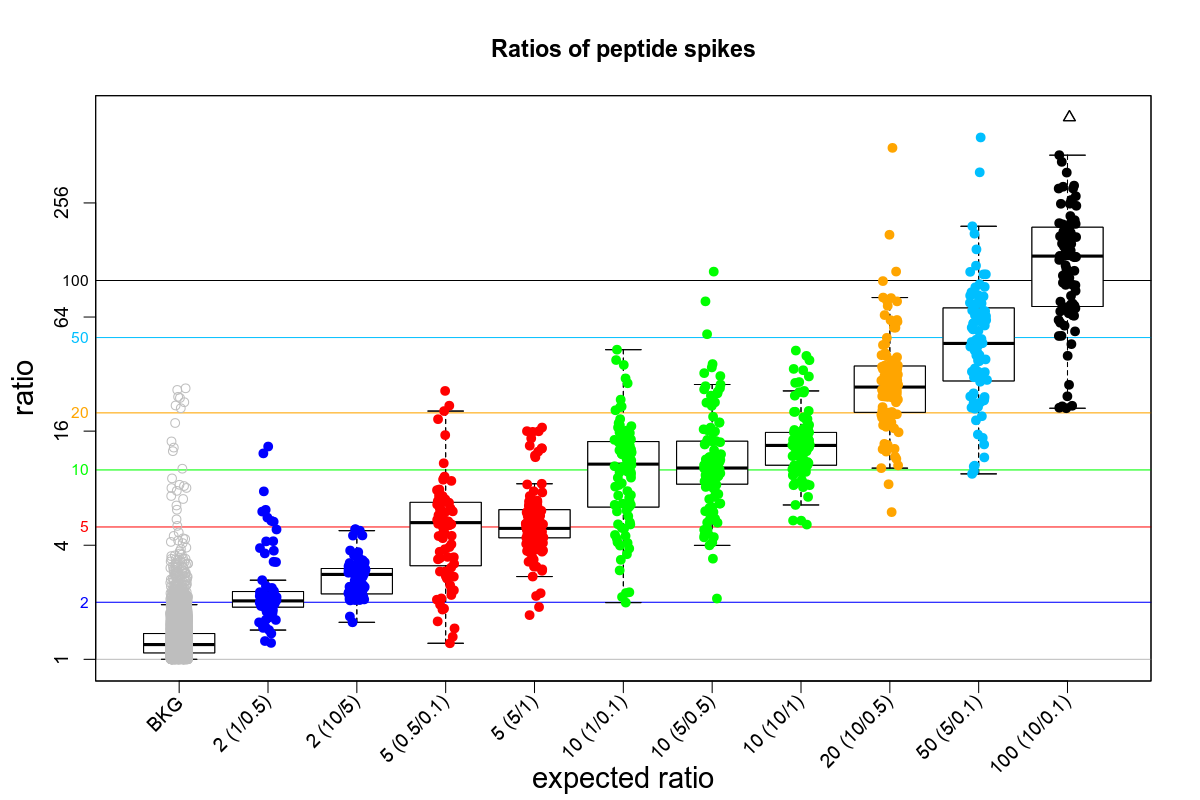

Supplement: Supplemental file 03 - additional plots [file 155027_1_supp_412484_pzgndk.zip › accuracy_plots/05-GS_IS_30-peptides.png]

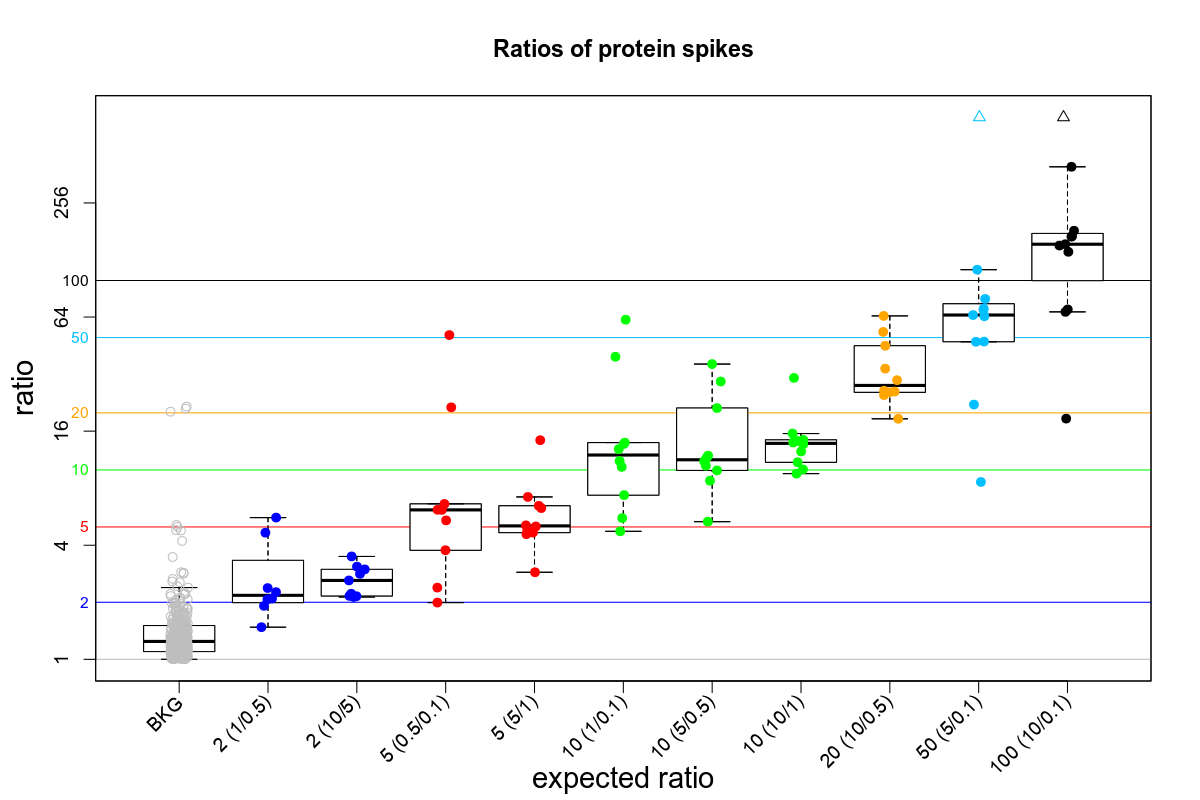

Supplement: Supplemental file 03 - additional plots [file 155027_1_supp_412484_pzgndk.zip › accuracy_plots/05-GS_IS_30-proteins.png]

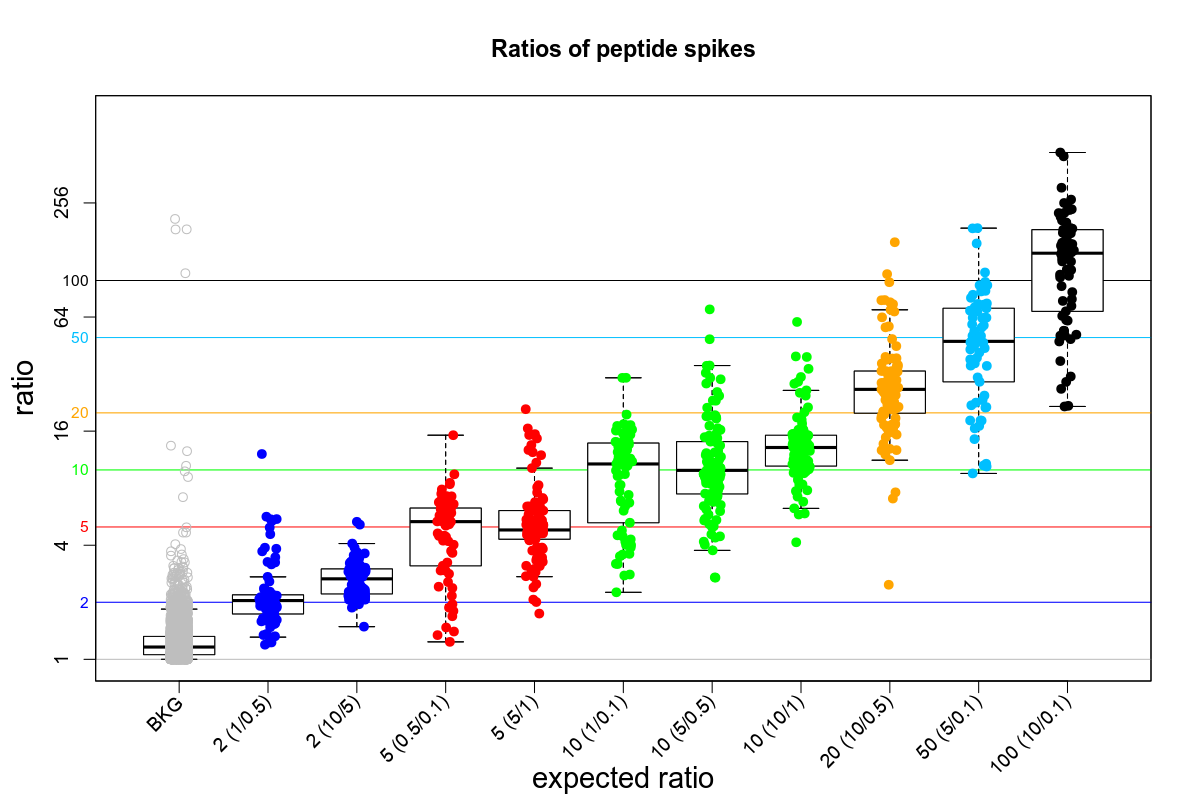

Supplement: Supplemental file 03 - additional plots [file 155027_1_supp_412484_pzgndk.zip › accuracy_plots/06-MM_F_Prot_20-peptides.png]

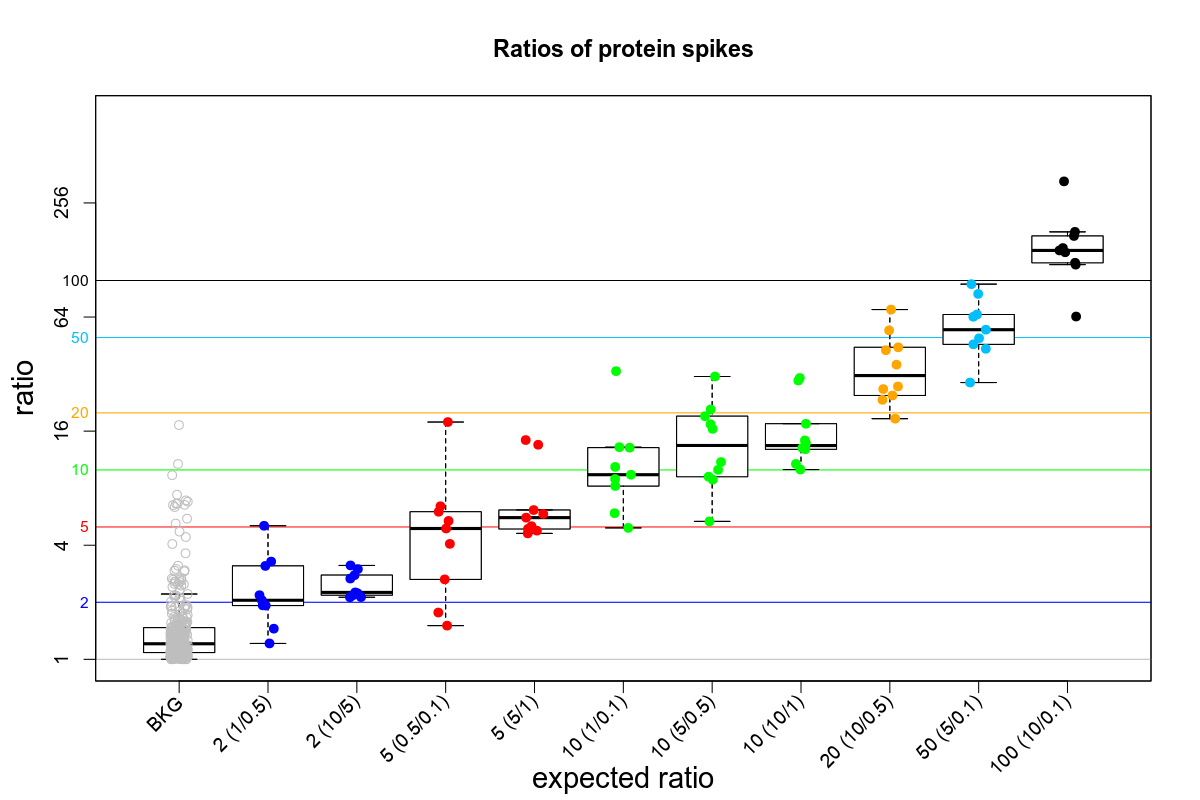

Supplement: Supplemental file 03 - additional plots [file 155027_1_supp_412484_pzgndk.zip › accuracy_plots/06-MM_F_Prot_20-proteins.png]

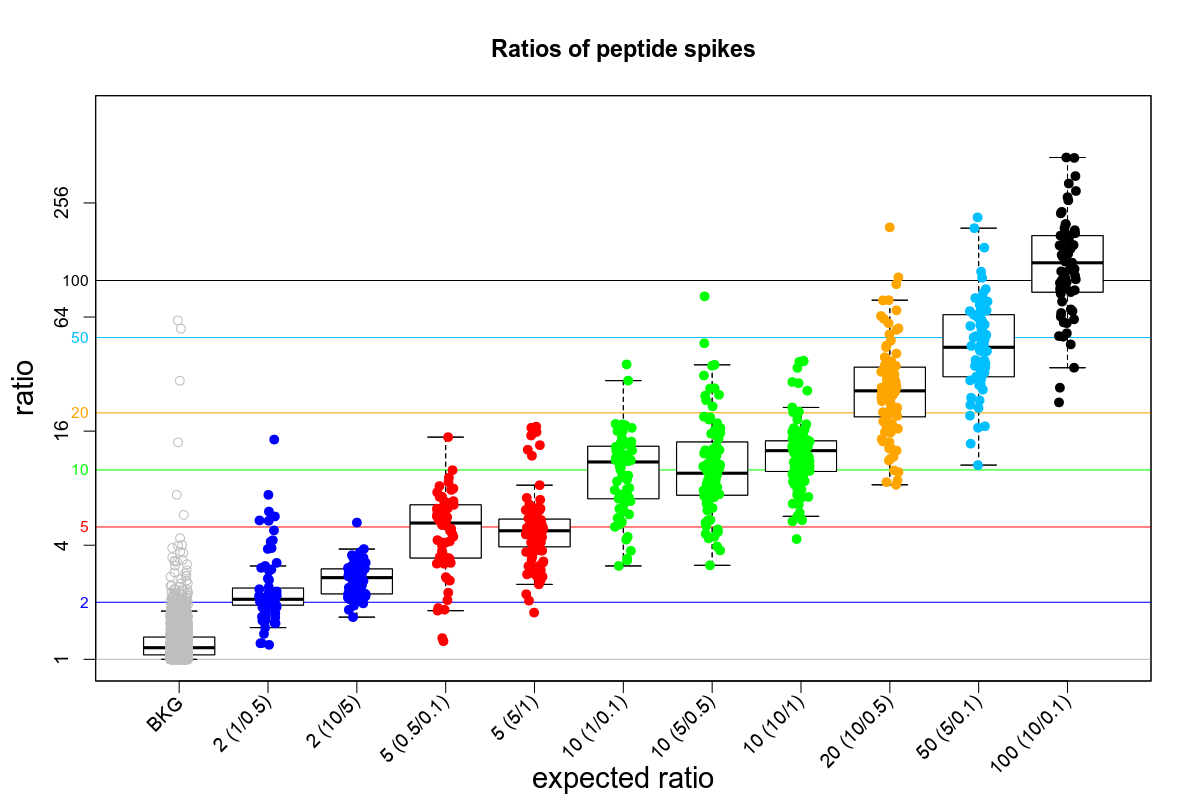

Supplement: Supplemental file 03 - additional plots [file 155027_1_supp_412484_pzgndk.zip › accuracy_plots/07-MM_F_Pep_16-peptides.png]

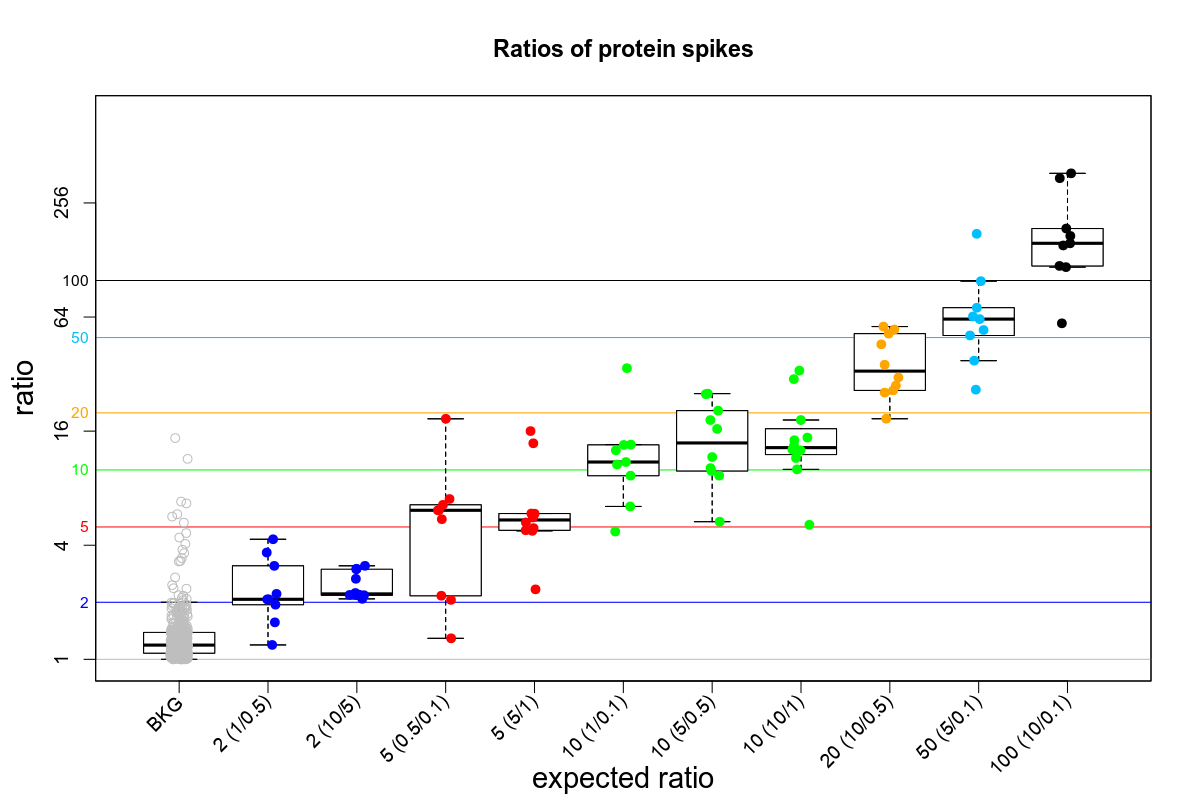

Supplement: Supplemental file 03 - additional plots [file 155027_1_supp_412484_pzgndk.zip › accuracy_plots/07-MM_F_Pep_16-proteins.png]

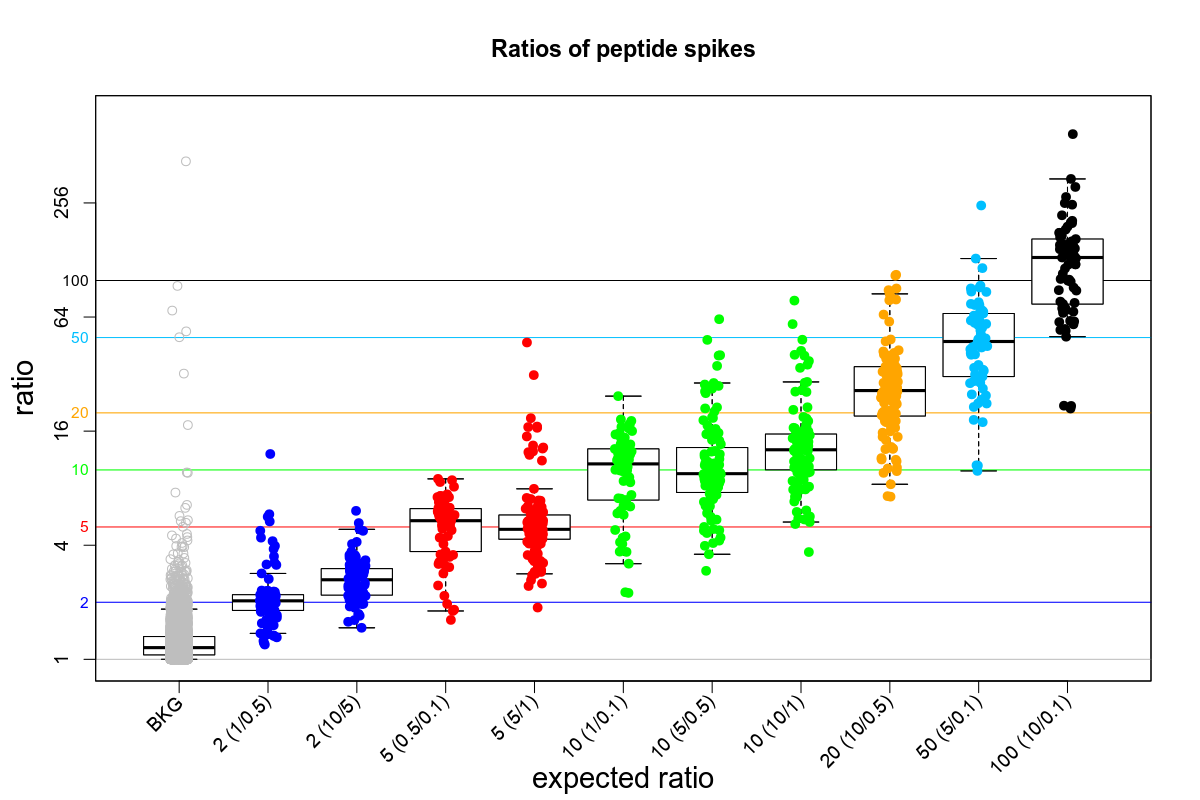

Supplement: Supplemental file 03 - additional plots [file 155027_1_supp_412484_pzgndk.zip › accuracy_plots/08-IS_and_F_all_78-peptides.png]

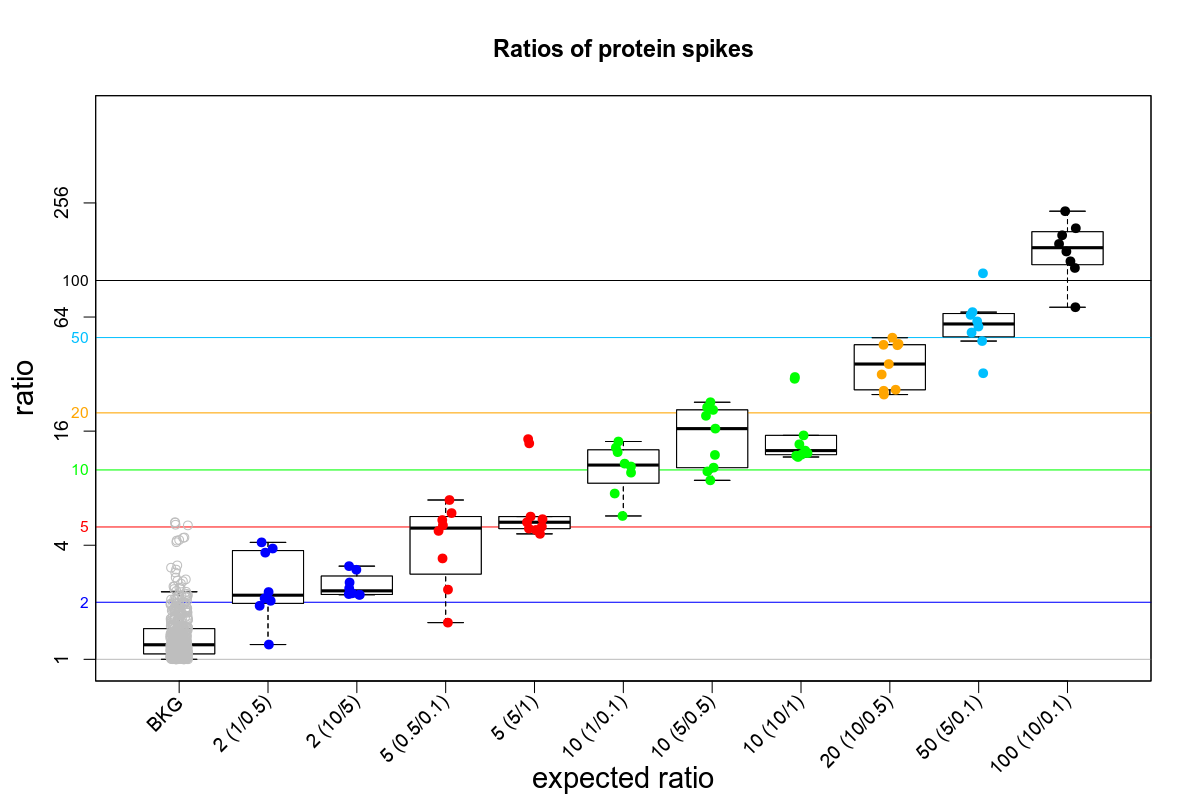

Supplement: Supplemental file 03 - additional plots [file 155027_1_supp_412484_pzgndk.zip › accuracy_plots/08-IS_and_F_all_78-proteins.png]

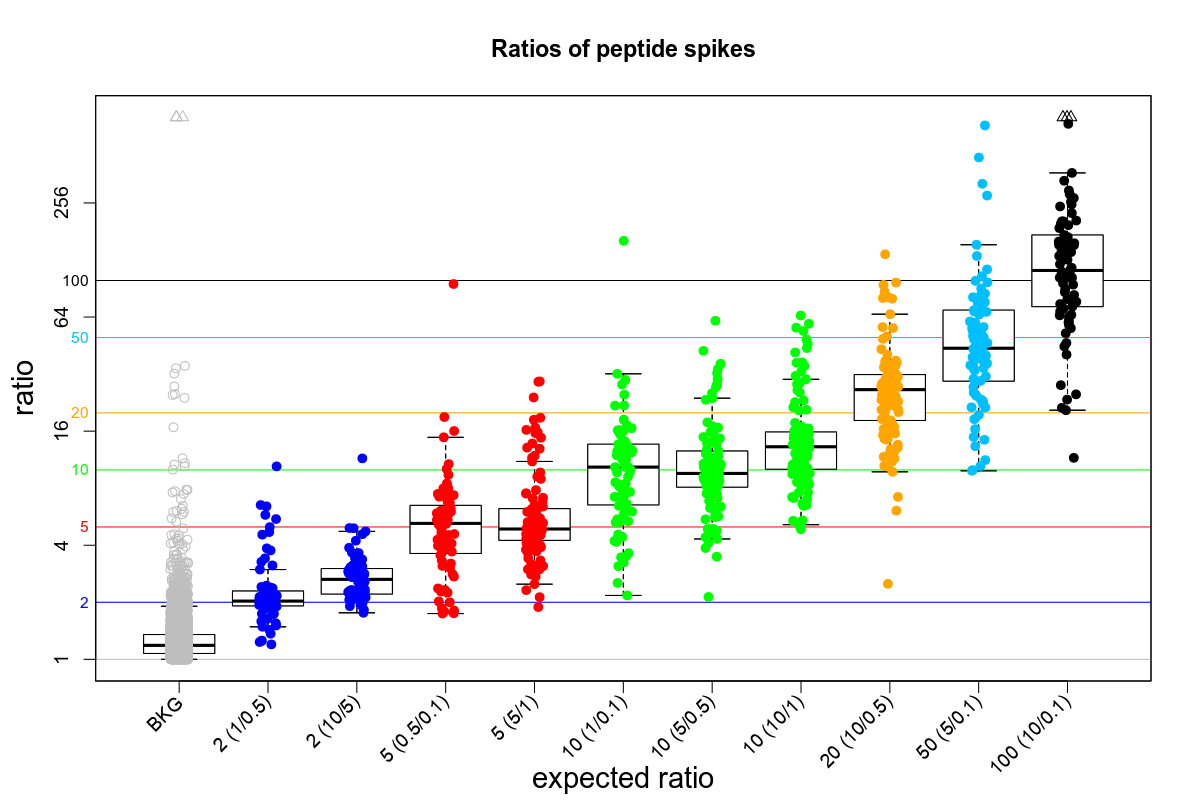

Supplement: Supplemental file 03 - additional plots [file 155027_1_supp_412484_pzgndk.zip › accuracy_plots/09-Pulsar_GS_IS_15-peptides.png]

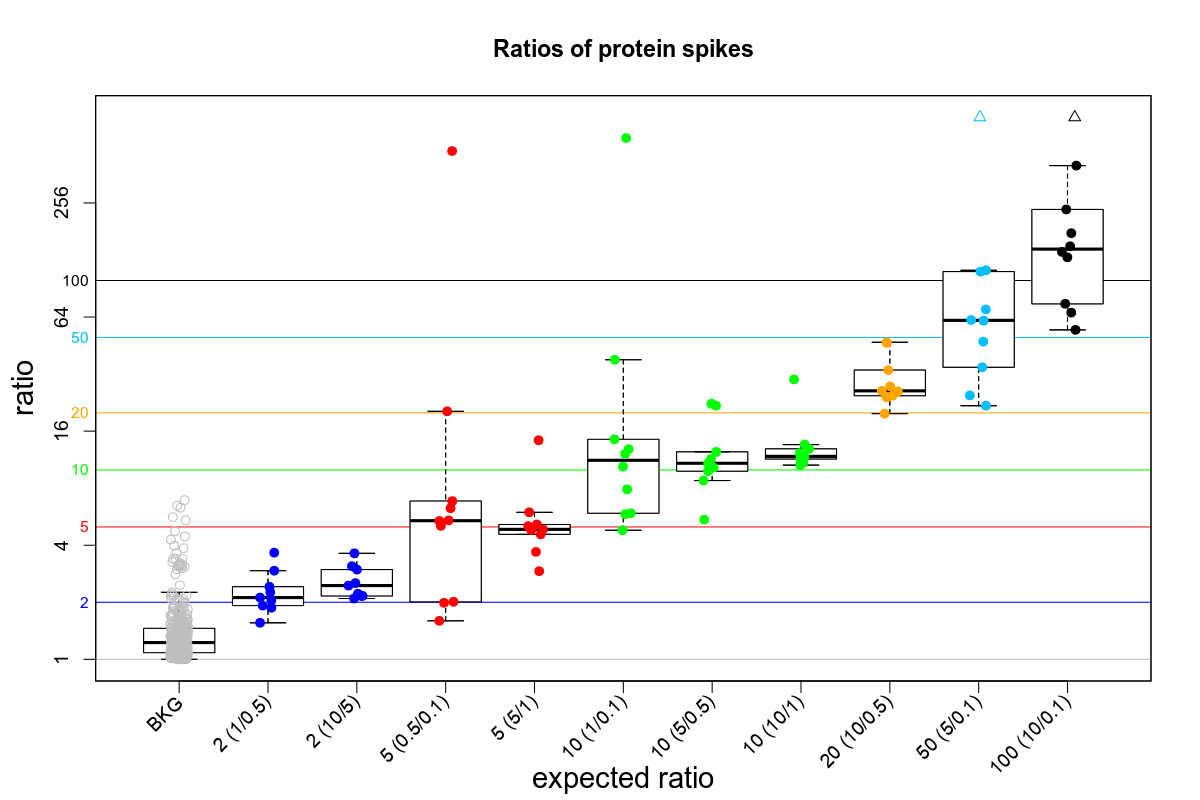

Supplement: Supplemental file 03 - additional plots [file 155027_1_supp_412484_pzgndk.zip › accuracy_plots/09-Pulsar_GS_IS_15-proteins.png]

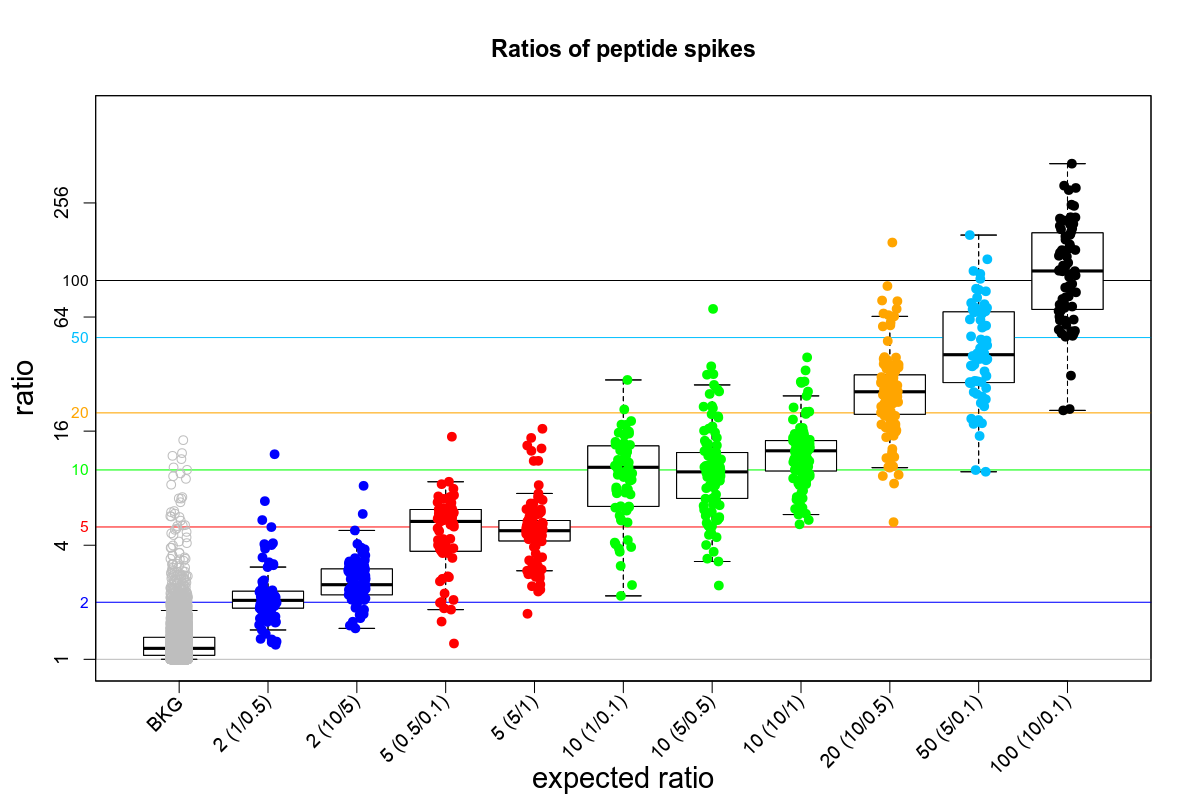

Supplement: Supplemental file 03 - additional plots [file 155027_1_supp_412484_pzgndk.zip › accuracy_plots/10-Pulsar_MM_F_Prot_20-peptides.png]

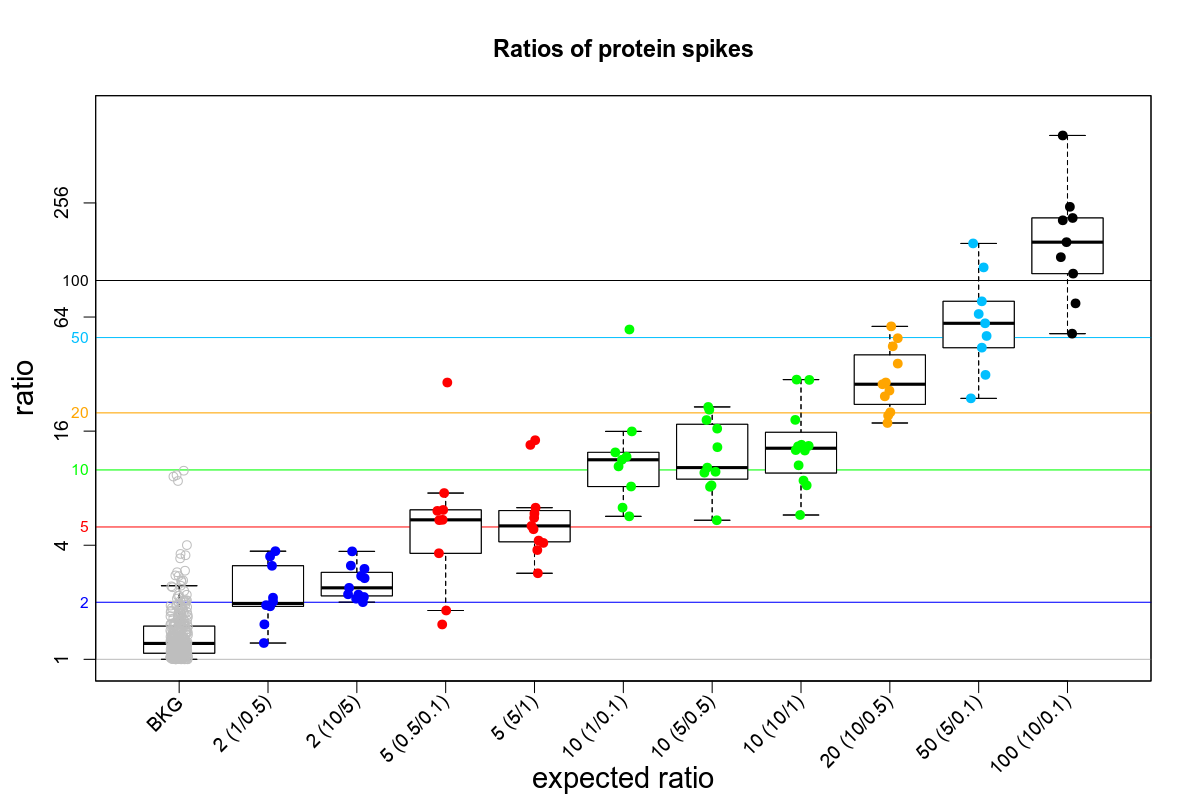

Supplement: Supplemental file 03 - additional plots [file 155027_1_supp_412484_pzgndk.zip › accuracy_plots/10-Pulsar_MM_F_Prot_20-proteins.png]
